# Heavy metals and neurodevelopment of children in low and middle-income countries: A systematic review

**DOI:** 10.1371/journal.pone.0265536

**Published:** 2022-03-31

**Authors:** Yi Yan Heng, Iqra Asad, Bailey Coleman, Laura Menard, Sarah Benki-Nugent, Faridah Hussein Were, Catherine J. Karr, Megan S McHenry

**Affiliations:** 1 Department of Pediatrics, Indiana University School of Medicine, Indianapolis, Indiana, United States of America; 2 School of Science, Indiana University-Purdue University Indianapolis, Indianapolis, Indiana, United States of America; 3 School of Health and Human Sciences, Indiana University-Purdue University Indianapolis, Indianapolis, Indiana, United States of America; 4 Ruth Lilly Medical Library, Indiana University School of Medicine, Indianapolis, Indiana, United States of America; 5 Department of Global Health, University of Washington, Seattle, Washington, United States of America; 6 Department of Chemistry, College of Biological and Physical Sciences of the University of Nairobi, Nairobi, Kenya; 7 Department of Environmental and Occupational Health Sciences, Department of Pediatrics, University of Washington, Seattle, Washington, United States of America; US Environmental Protection Agency, UNITED STATES

## Abstract

**Background:**

The presence of harmful environmental exposures, which disproportionately affects low-and-middle income countries (LMICs), contributes to >25% of deaths and diseases worldwide and detrimentally affects child neurodevelopment. Few resources succinctly summarize the existing literature on this topic. Our objective is to systematically review and characterize the evidence regarding the relationship between heavy metals and neurodevelopment of children in LMICs.

**Methods:**

We conducted a medical librarian-curated search on multiple online databases to identify articles that included individuals <18 years living in a LMIC, quantitatively measured exposure to a heavy metal (either prenatal or postnatal), and used a standardized measurement of neurodevelopment (i.e. cognitive, language, motor, and behavior). Reviews, editorials, or case studies were excluded. Results were analyzed qualitatively, and quality was assessed.

**Results:**

Of the 18,043 screened articles, 298 full-text articles were reviewed, and 100 articles met inclusion criteria. The included studies represented data from 19 LMICs, only one of which was classified as a low-income country. Ninety-four percent of postnatal lead and all postnatal manganese studies showed a negative association with metal exposure and neurodevelopment, which were the strongest relationships among the metals studied. Postnatal exposure of mercury was associated with poor neurodevelopment in only half of studies. Limited data on postnatal arsenic and cadmium suggests an association with worse neurodevelopment. Findings were mixed for prenatal arsenic and lead, although some evidence supports that the neurotoxicity of lead was amplified in the presence of manganese.

**Conclusions and potential impact:**

We found that lead and manganese appear to consistently have a detrimental effect on the neurodevelopment of children, and more evidence is needed for mercury, arsenic, and cadmium. Better characterization of these effects can motivate and inform prioritization of much needed international policies and programs to reduce heavy metal exposures for young children within LMICs.

## 1. Introduction

Harmful environmental exposures contribute to approximately 25% of deaths and diseases worldwide [1]. The burden of these exposures disproportionately impacts low- and middle-income countries (LMICs). This is due to limited resources and insufficient infrastructure in LMICs to regulate and minimize toxicant exposures, leading to significantly higher levels of environmental pollutants [2, 3]. These harmful environmental exposures compound the many risk factors and challenges in healthcare systems faced by children living in LMICs, resulting in worse health outcomes [4].

Heavy metals are a common harmful environmental exposure within these settings and are frequently found to exceed the recommended levels. Currently, an estimated 632 million children living in LMICs have a blood lead level greater than the US Centers for Disease Control’s prior public health action level of 5 μg/dL [5], which was updated in 2021 to be lowered to 3.5 μg/dL [6]. Over 50 countries, including Bangladesh, Cambodia, Egypt, and Ghana, have levels of manganese exceeding 400 μg/L in their drinking water. Although the World Health Organization (WHO) no longer provides guidelines on manganese [7], studies have shown that high levels are associated with various detrimental impacts such as depressed intellectual function in children [8]. In Latin America, over 4.5 million people are chronically exposed to arsenic levels in their drinking water that are more than 200 times higher than the WHO stipulated limit of 10 μg/L [9]. Many of these heavy metals result from poorly regulated use in agriculture, mining, smelting, illegal refining, and industrial production; and they contaminate the environment through air, water, and soil [10]. Since they are non-biodegradable, environmental concentrations can build up slowly over time. Thus, cumulative exposures in human populations can have long-lasting negative impacts [4].

Children are particularly susceptible to the detrimental health effects of environmental exposures due to their rapid neurodevelopment. During the first 1000 days of life, the brain has maximal plasticity and is transformed through a series of intricate events, such as neurogenesis, myelination, synaptic pruning, among others [11]. These events build upon one another and, over time, manifest as vital cognitive functions, such as language and speech production, attention, behavior, and reasoning [12]. Thus, disruptions in their biological environments, including exposure to toxicants, may disturb the fine orchestration of events, causing irreversible downstream effects such as neurodevelopmental delays, behavioral problems, and learning disabilities [13]. In addition to this period of rapid brain plasticity, other biological and social factors put children at increased risk of harmful environmental exposures as compared to the adults. Children consume more food and water for their body weight, spend extended time on the ground and floor, and tend to put more non-food items in their mouth [14].

The detrimental effects on heavy metals on child neurodevelopment have been well-studied within high-income countries. The heavy metal with the most evidence of neurodevelopmental detriment is lead, with robust evidence for a decreased IQ scores, in a dose-dependent manner [15, 16]. In their review, the National Toxicology Program has deemed the evidence sufficient for adverse effects of lead on cognition as well as enhanced attention related and other problem behaviors, even for blood lead levels below 5 ug/dL [17]. Likewise, direct exposure to other metals, such as arsenic, cadmium, manganese, and mercury, have been shown to have detrimental effects on various aspects of neurodevelopment, such as cognition and behavior [8, 17–26]. Inconclusive evidence exists regarding the potential effect of prenatal arsenic exposure on neurodevelopment [27, 28]. However, the majority of studies examining relationships between heavy metals and child neurodevelopment occur in well-resourced settings; and not within LMICS, where the 90% of the world’s children live [29].

Currently, an estimated 43% of children in LMICs do not reach their full neurodevelopmental potential due to a myriad of factors, including environmental exposures [30]. This impacts their quality of life and economic potential [11] and highlights the critical need to understand the impact of heavy metals on neurodevelopment within these settings. In doing this, we may better inform and prioritize future research and policy to optimize outcomes for children in LMICs. The objective of this study was to systematically review and summarize the evidence on exposure to heavy metals and its association with child neurodevelopmental outcomes in LMICs.

## 2. Materials and methods

The Preferred Reporting Items for Systematic review and Meta-Analysis (PRISMA) Protocols 2015 Checklist was used when conducting this systematic review [31]. The general systematic review protocol was registered with PROSPERO on April 28, 2020 (CRD42020166245). In August 2020, an amendment was submitted noting that the exclusion criteria were further refined to exclude studies from high-income countries and the review was split into three focused topic areas: heavy metals, pesticides, and air pollution. The latter two reviews will be described elsewhere.

### 2.1. Eligibility criteria

The following inclusion criteria were applied: (1) included individuals < 18 years of age for whom used a standardized neuropsychological test was performed (2) quantitatively measured exposure to an environmental heavy metal in either child or the mother while child was in utero, (3), was conducted in a low-and-middle income country, as determined by using the World Bank Criteria (low-income defined as a gross national income per capita of $1,045 or less in 2020, lower-middle as those between $1,046 and $4,095, and upper-middle as those between $4,096 and $12,695) [32], (4) used either an observational cohort, cross-sectional, quasi experimental, or ecological study design, and (5), were conducted after 1970. Only full-text articles published in English were included. Review articles, published abstracts, case studies, and studies without cognitive or behavioral functioning for each subject were excluded. Certain metals that are not traditionally viewed as harmful heavy metals, such as zinc, were excluded.

### 2.2. Search strategy

A medical librarian worked with M.S.M. to create a combination of MeSH and keywords pertaining to the problem/population, intervention, and setting, which were used when conducting the search. Among the MeSH terms used in this search, we included “Metals, Heavy”, which encompassed the metals actinium, americium, antimony, barium, berkelium, bismuth cadmium, californium, cesium, cesium isotopes, chromium, chromium isotopes, cobalt, copper, curium, einsteinium, fermium, francium, gallium, gallium isotopes, germanium, gold, gold isotopes, hafnium, indium, iridium, iron, iron isotopes, lawrencium, lead, manganese, mendelevium, mercury, mercury isotopes, molybdenum, neptunium, nickel, niobium, nobelium, osmium, palladium, platinum, plutonium, protactinium, radium, rhenium, rhodium, rubidium, ruthenium, and silver (see S1 File for full search strategy details). The following databases were searched last on 17 March 2021: PubMed MEDLINE, Embase, Cochrane Library, CINAHL, PsychInfo, Scopus, and Web of Science. Bibliographies of pertinent articles were also hand searched. Records were managed by the screening and data extraction tool Covidence [33] and the citation managing program Zotero [34]. The initial screening was performed by two independent reviewers (I.A. and B.C.) who each assessed all of the titles and abstracts from the search and excluded any articles that did not meet the inclusion criteria. The full-text screening was then completed on all articles by two independent reviewers (Y.H. and either I.A. or B.C). Each paper was screened by two individuals, both in the initial screening and the full-text screening. Disagreements between the 2 reviewers were settled by a fourth reviewer (M.S.M.)

### 2.3. Data extraction

Three reviewers (Y.H., I.A., and B.C.) divided up the articles, so that between the three of them, each article was independently extracted twice. Discrepancies in extraction were discussed and the original articles were reviewed to confirm details and obtain consensus among the group. The following variables were extracted from the studies: study population (country, sample size, age at outcome measurement, whether the study was conducted in utero, exposure type and levels), study design, neuropsychological domain tested (cognitive, language, motor, and behavior), and results. Studies were defined as “prenatal” if at least one component of the exposure was measured while the child was in utero.

### 2.4. Quality assessment

The quality of these studies was independently assessed by Y.H. and either I.A. or B.C. using the NIH Study Quality and Assessment Tools [35], so that each article was independently read by 2 of the authors. These tools were developed by the National Heart, Lung, and Blood institute as a way for reviewers to assess studies’ internal validity. While useful, these guidelines do not contain standardized metrics for how to rate the overall quality of each study. Thus, Y.H., I.A., and B.C., jointly determined their parameters for making judgements. There were 3 general categories of ratings: good, fair, or poor. For cross-sectional studies, scores 9/14 and above were rated “good”, scores 7/14 and 8/14 were rated “fair”, and all other scores were rated “poor”. For case control studies, scores 9/12 and above were rated “good”, scores 7/12 and 8/12 were rated “fair”, and all other scores were rated “poor”. Each criterion was given the same weight. If ratings differed, the reviewers conferred to reach a consensus.

### 2.5. Data synthesis and analysis

The data available were not amenable to a meta-analysis, due to the heterogeneity of the exposure assessment methodologies and level thresholds, preventing a direct comparison. Thus, we performed a qualitative summarization of the data. For articles that examined multiple mixed metal exposures, only the interactions among the metals were summarized within the results of this review. However, full summaries of all mixed metal articles are included in S1 Table.

## 3. Results

### 3.1. Description of search results

**Fig 1:**
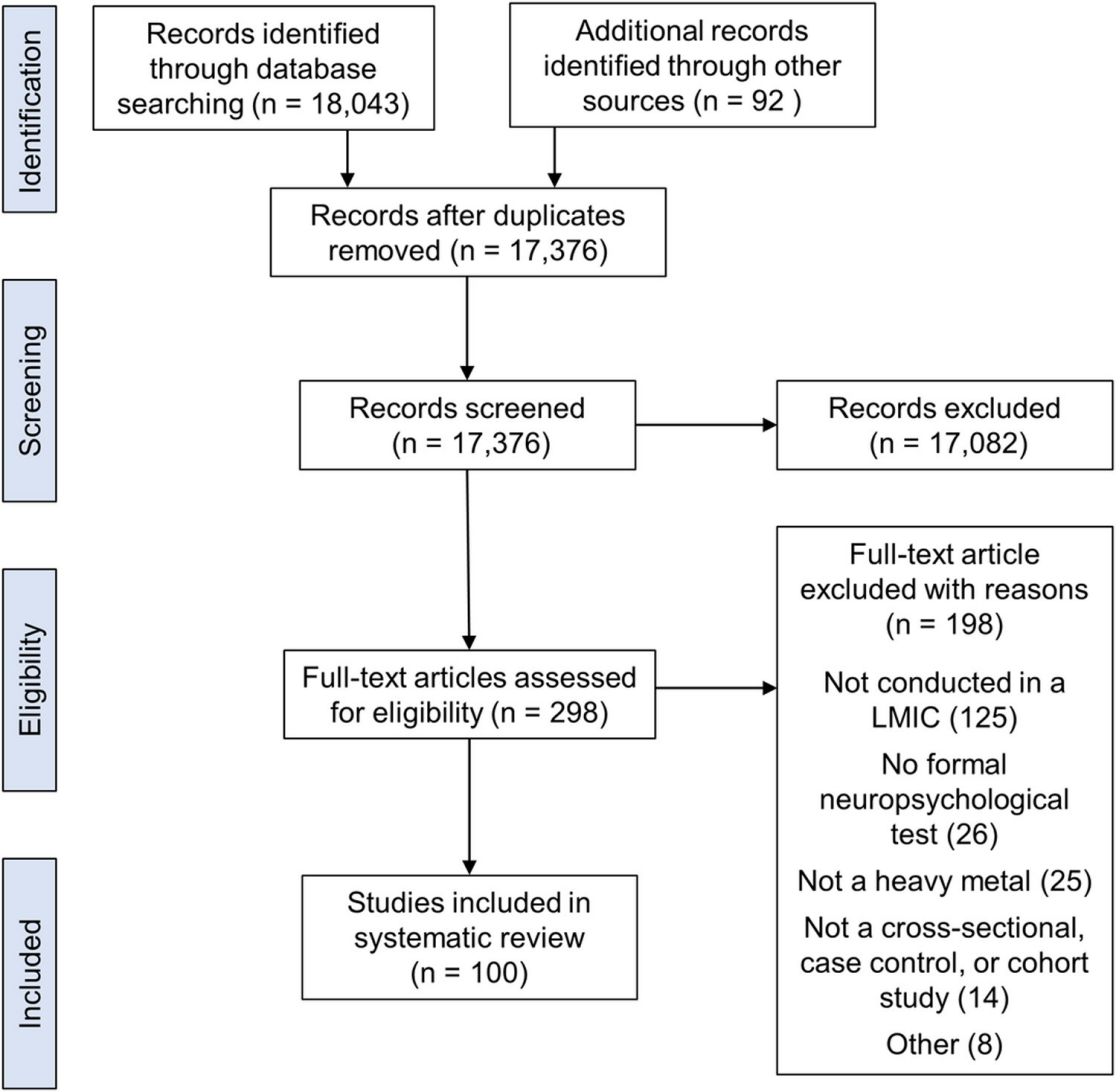
PRISMA diagram.

The search strategy yielded a total of 17,376 records (after duplicates were removed). After the initial screen, 298 studies remained for full-text article review. One hundred publications met criteria for inclusion in our systematic review (Fig 1).

### 3.2. Study characteristics

#### 3.2.1. General characteristics

**Fig 2:**
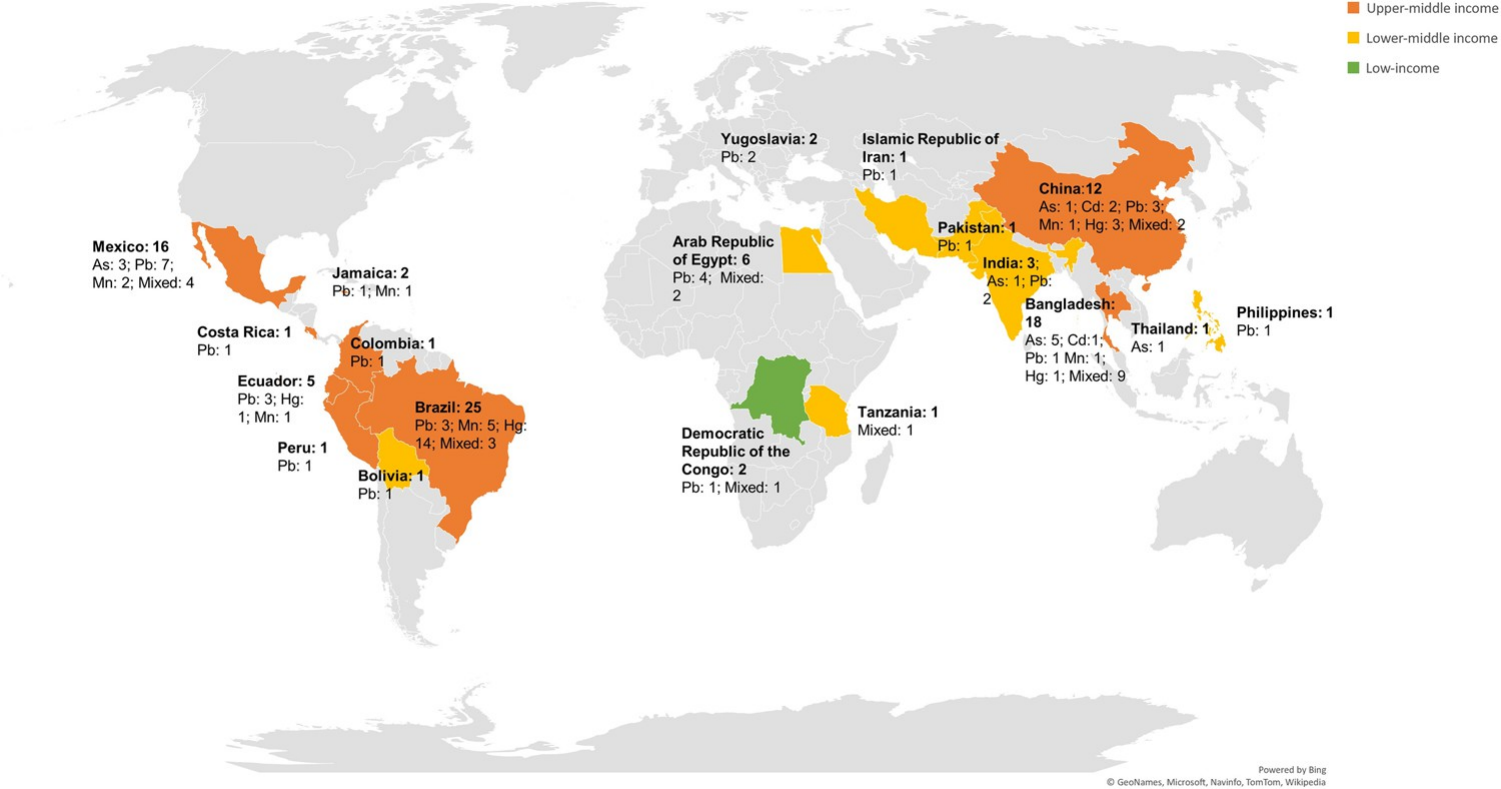
Countries included in this review.

The majority of the studies (n = 64) were conducted in upper-middle income countries (Brazil, Colombia, Costa Rica, Ecuador, Jamaica, Mexico, Peru, China, Thailand), as defined by the World Bank [32]. Thirty-two studies were conducted in countries with lower-middle income economies (the Arab Republic of Egypt, Bangladesh, Bolivia, India, the Islamic Republic of Iran, Pakistan, Philippines, and Tanzania), and 2 studies were conducted in a country with a low-income level (the Democratic Republic of the Congo). The country formerly known as Yugoslavia was included but not given a specific income classification. Seven cohorts, based primarily in Latin America and Bangladesh, contributed data to 24 studies included within this review (Fig 2).

The studies within this review addressed 5 heavy metals, lead (n = 34), mercury (n = 19), arsenic (n = 11), manganese (n = 11), and cadmium (n = 3), In this review, twenty-two studies analyzed multiple metals concurrently. The most common study design was cross-sectional (n = 62); 34 were prospective cohort studies, and 4 were case-control studies. Of the 100 publications studied, 18 measured heavy metals prenatally, in which measurements of heavy metals were taken before birth, and 82 measured heavy metals postnatally, in which measurements of heavy metals were only taken after birth. Of the prenatal studies, 8 studies only measured heavy metal levels prenatally, while 10 studies also measured heavy metals at least one other time during development. Eighty-eight articles were rated as being good quality, 11 were fair, and one was poor.

#### 3.2.2. Methods of outcome assessment

Neurodevelopmental domains that were analyzed broadly included intelligence, cognition, and behavior. The most frequently used assessments were various editions and subtests of the Weschler’s Intelligence Scale for Children (WISC) (n = 33), the Bayley Scales of Infant Development (BSID) (n = 18), the Gesell Developmental Scale (GDS) (n = 8), the Raven’s Progressive Matrices (n = 10), and the Child Behavior Checklist (CBCL) (n = 5). These neurodevelopment assessments were performed at a variety of ages: 20 studies assessed children under age one year, 43 assessed children between ages 1–5 years, 58 assessed children between ages 6–12 years, and 13 assessed adolescents > 12 years. When examining the age of outcome assessment for each heavy metal exposure and whether an association existed, no clear trends were found.

### 3.3. Arsenic

#### 3.3.1. Study characteristics

The 11 studies on arsenic were conducted in Bangladesh (n = 5), China (n = 1), India (n = 1), Mexico (n = 3), and Thailand (n = 1). These studies were published between 1999 and 2020 and examined outcomes for children ranging from 7 months to 15 years of age. Five studies were prospective cohort studies that examined arsenic exposure prenatally; among these studies, 4 also evaluated concurrent arsenic exposure at neurodevelopmental assessment. 6 others were cross-sectional studies that examined arsenic exposure at the same time as postnatal neurodevelopment assessments. These studies involved a total of 18,582 participants that ranged from 132 to 7479 participants per study. Two of the studies also measured and controlled for lead exposure. All quality assessments of the studies were rated “good.” Additional details about these studies can be found in Tables 1–3, and S1.

**Table 1:** Studies that found a negative association between heavy metal exposure and various domains of neurodevelopment.

Heavy metal exposure	Infant development	Behavior	Intelligence	Other domains of neurodevelopment
Arsenic				
Prenatal exposure			Hamadani et al. (2011) [36], Vahter et al. (2020) [37]	
Postnatal exposure			Hamadani et al. (2011) [36], Rocha-Amador et al. (2007) [38], Siripitayakunkit et al. (1999) [39], Wang et al. (2007) [40]	Asadullah and Chaudhury (2011) [41], Rosado et al. (2007) [42], von Ehrenstein et al. (2007) [43]
Cadmium				
Prenatal exposure		Wang et al. (2016) [44]	Tian et al. (2009) [45]	
Postnatal exposure		Gustin et al. (2018) [46]	Gustin et al. (2018) [46]	
Lead				
Prenatal exposure	Vigeh et al. (2014) [47]	Wasserman et al. (1998) [48]	Schnaas et al. (2000) [49]	
Postnatal exposure	Braun et al. (2012) [50], Gleason et al. (2020) [51], Kordas et al. (2011) [52], Schnaas et al. (2000) [49], Solon et al. (2008) [53], Téllez-Rojo et al. (2006) [54], Wasserman et al. (1998) [48]	AbuShady et al. (2017) [55], Kashala-Abotnes et al. (2016) [56], Olympio et al. (2010) [57], Rahman et al. (2002) [58], Vega-Dienstmaier et al. (2006) [59]	Alvarez-Ortega et al. (2017) [60], Bellinger et al. (2005) [61], Counter et al. (1998) [62], Counter et al. (2005) [63], Kamel et al. (2003) [64], Liu et al. (2013) [65], Mostafa et al. (2009) [66], Rahman et al. (2002) [58], Rasoul et al. (2012) [67], Schnaas et al. (2006) [68], Solon et al. (2008) [53], Wang et al. (1989) [69], Wang et al. (2009) [70], Wasserman et al. (2003) [71]	Kamel et al. (2003) [64], Kordas et al. (2006) [72], Malavika et al (2021) [73], Liu et al. (2013) [65], Rahman et al. (2002) [58], Counter et al. (2008) [74], Kordas et al. (2004) [75], Vega-Dienstmaier et al. (2006) [59], do Nascimento et al. (2014) [76]
Manganese				
Prenatal exposure		Rahman et al. (2017) [77]		
Postnatal exposure	Claus Henn et al. (2010) [78], Yu et al. (2014) [79]	Carvalho et al. (2018) [80], Menezes-Filho et al. (2014) [81], Rahman et al. (2017) [77]	Βetancourt et al. (2015) [82], Carvalho et al. (2014) [83], Menezes-Filho et al. (2011) [84]	Carvalho et al. (2018) [80], Nascimento et al (2016) [85], Rahbar et al. (2014) [86], Torres-Agustín et al. (2013) [87]
Mercury				
Prenatal exposure	Rothenberg et al. (2021) [88]			
Postnatal exposure	Dórea et al. (2012) [89], Gao et al. (2007) [90], Marques et al. (2016) [91], Marques et al. (2009) [92], Marques et al. (2010) [93], Marques et al. (2015) [94]		Grandjean et al. (1999) [95]	Chevrier et al. (2009) [96], Counter et al. (2006) [97], Grandjean et al. (1999) [95]

**Table 2:** Studies that found no association between heavy metal exposure and various domains of neurodevelopment.

Heavy metal exposure	Infant development	Behavior	Intelligence	Other domains of neurodevelopment
Arsenic				
Prenatal exposure	Hamadani et al. (2010) [98], Tofail et al. (2009) [99]		von Ehrenstein et al. (2007) [43]	
Postnatal exposure		Hamadani et al. (2010) [98], Roy et al. (2011) [100]	Vahter et al. (2020) [37]	Rosado et al. (2007) [42]
Cadmium				
Prenatal exposure		Gustin et al. (2018) [46]	Wang et al. (2016) [44], Gustin et al. (2018) [46]	
Postnatal exposure				
Lead				
Prenatal exposure				
Postnatal exposure	Kashala-Abotnes et al. (2016) [56], Wolf et al. (1994) [101]	Rahman et al. (2002) [58], Wang et al. (2009) [70], Wolf et al. (1994) [101]	Liu et al. (2013) [65], Solon et al. (2008) [53], Wolf et al. (1994) [101]	AbuShady et al. (2017) [55], Gahyva et al. (2008) [102], Kordas et al. (2004) [75], Kordas et al. (2006) [72], Rahbar et al. (2015) [103]
Manganese				
Prenatal exposure			Rahman et al. (2017) [77]	
Postnatal exposure	Claus Henn et al. (2010) [78]		Carvalho et al. (2014) [83], Menezes-Filho et al. (2011) [84]	Torres-Agustín et al. (2013) [87]
Mercury				
Prenatal exposure				
Postnatal exposure	Dórea et al. (2014) [104], Marques et al. (2007) [105], Marques et al. (2016) [106]		Fonseca et al. (2008) [107], Gao et al. (2007) [90], Marques et al. (2016) [108]	Tavares et al. (2005) [109]

**Table 3:** Studies that found positive associations between heavy metal exposures and various domains of neurodevelopment.

Heavy metal exposure	Infant development	Behavior	Intelligence	Other domains of neurodevelopment
Arsenic				
Prenatal exposure				
Postnatal exposure				
Cadmium				
Prenatal exposure				
Postnatal exposure				
Lead				
Prenatal exposure	Ruiz-Castell et al. (2012) [110]			
Postnatal exposure				Wolf et al. (1994) [101]
Manganese				
Prenatal exposure				Rahman et al. (2017) [77]
Postnatal exposure				
Mercury				
Prenatal exposure	Hu et al. (2016) [111]			
Postnatal exposure	Marques et al. (2012) [112], Marques et al. (2016) [108]	Gustin et al. (2017) [113]		

#### 3.3.2. Exposure assessment methods for arsenic

Of the studies that measured arsenic exposure, 8 measured arsenic levels in urine [36, 37, 40, 42, 43, 98–100], 4 measured arsenic levels in water [38, 40, 41, 43], 2 studies measured arsenic levels in hair [37, 39], and one study measured arsenic levels in blood [37].

#### 3.3.3. Maternal arsenic exposure during pregnancy and child neurodevelopment

Of the 5 studies that measured prenatal arsenic levels, 3 reported no significant association between arsenic levels and neurodevelopment [43, 98, 99], and 2 found adverse associations [36, 37]. One study reported that maternal arsenic levels in urine during pregnancy was negatively associated with the Verbal Intelligence Quotient (VIQ) and Full Scale Intelligence Quotient (FSIQ) for girls only [36], and the other reported that children with maternal urinary arsenic levels between 122 and 246 μg/L at early pregnancy had lower FSIQ scores compared to those with maternal urinary levels between 3.2 and 32.4 μg/L. However, there was no significant difference between IQ scores of children with maternal urinary arsenic levels > 247 μg/dL. Arsenic levels later in the pregnancy had no significant effect on IQ scores [37].

#### 3.3.4. Postnatal exposure to arsenic and child neurodevelopment

Of the 10 studies that analyzed postnatal arsenic levels and neurodevelopment, 8 reported a negative association between arsenic levels and at least one domain of neurodevelopment, such as, but not limited to executive functioning (e.g. working memory, processing speed, attention, inhibition), general cognition, language, fine motor, or gross motor domains [36–43], and 2 reported no significant effect [98, 100].

*3*.*3*.*4*.*1*. *Intelligence*. Four studies reported that elevated arsenic levels were associated with worse IQ scores [36, 38–40]. One study, however, reported no significant difference in IQ score in children with arsenic levels > 163 μg/L [37].

*3*.*3*.*4*.*2*. *Other domains of neurodevelopment*. One study reported worse math test scores in primary and secondary schools for children with arsenic-poisoned home wells compared to those with arsenic-free wells [41]. Another study reported a negative association between urinary arsenic levels and several tests: the Visual-Spatial Abilities with Figure Design, the Peabody Picture Vocabulary Test, the WISC-RM Digit Span Subtest, Visual Search, and Letter Sequencing, but no significant association with math achievement, the Wechsler Intelligence Scale for Children, Revised Mexican Version (WISC-RM) Arithmetic Subtest, Sternberg Memory, Visual Memory Span, WISC-RM Coding Subtests, and Stimulus Discrimination [42]. One study showed that compared to children with < 43 μg/dL of urinary arsenic, those with urinary arsenic levels > 82.6 μg/dL had a decline of 12% on the vocabulary, 21% on the object assembly, and 13% in the picture completion subtests of the WISC [43]. This study also reported results on prenatal exposure to arsenic, as noted above. Only 2 studies looked at behavioral domains with arsenic and no significant association was found [98, 100].

### 3.4. Cadmium

#### 3.4.1. Study characteristics

Three studies were conducted on cadmium in 2 different countries, including Bangladesh (n = 1) and China (n = 2). These studies were published between 2009 and 2018 and examined children ranging from 12 months to 10 years of age. All 3 studies were prospective cohort studies that examined prenatal cadmium levels; one of these studies also measured postnatal arsenic exposure at 10 years of age. These studies involved a total of 1708 participants that ranged from 106 to 1453 participants per study. One of the studies measured and controlled for lead, and 2 studies only analyzed cadmium. All quality assessments of the studies were rated “good.” Additional details about these studies can be found in Tables 1–3, and S1.

#### 3.4.2. Exposure assessment methods for cadmium

Of the 3 studies that measured cadmium exposure, one measured cadmium levels in urine [46] and 2 measured cadmium levels in blood [44, 45].

#### 3.4.3. Maternal cadmium exposure during pregnancy and child neurodevelopment

Two of the 3 studies showed a negative association between prenatal cadmium levels and at least one neurodevelopmental domain [44, 45], and one reported no significant effects [46]. One study reported that the performance IQ score and full scale IQ score was significantly lower in children with cord cadmium ≥ 6 μg/L compared with <0.6 μg/L [45]; the other study reported that a 10-fold increase in maternal cadmium level was associated with a 5.70 point decrease in the social domain of the GDS, not in VIQ [44]. One study, however, reported no significant difference between maternal urinary cadmium levels and scores on the WISC-IV and Strengths and Difficulties Questionnaire [46].

#### 3.4.4. Postnatal exposure to cadmium and child neurodevelopment

Only one study measured the effects of postnatal cadmium levels on neurodevelopment. This study showed a negative association between cadmium exposure and FSIQ scores, as children in the highest tertile of urinary cadmium measurements at 10 years scored 4.9 points lower than children in the lowest tertile. Furthermore, urinary cadmium levels were negatively associated with prosocial behavior and positively associated with hyperactivity in girls only [46].

### 3.5. Lead

#### 3.5.1. Study characteristics

The 34 studies were conducted in 17 different countries including Bangladesh (n = 1), Bolivia (n = 1), Brazil (n = 3), China (n = 3), Colombia (n = 1), Costa Rica (n = 1), Democratic Republic of the Congo (n = 1), Ecuador (n = 3), Arab Republic of Egypt (n = 4), India (n = 2), Islamic Republic of Iran (n = 1), Jamaica (n = 1), Mexico (n = 7), Pakistan (n = 1), Peru (n = 1), Philippines (n = 1), and the country formerly known as Yugoslavia (n = 2). These studies were published between 1989 and 2021 and examined children ranging from birth to 16 years. Twenty-five were cross-sectional studies, 4 were prospective cohort studies that measured lead exposure prenatally, 4 were prospective cohort studies that measured lead exposure at other time points, and one was a case control study. These studies involved a total of 9087 participants ranging from 20 to 1035 participants per study. Twenty-three studies were rated “good”, 8 were rated “fair”, and 1 was rated “poor” in quality. Additional details about these studies can be found in Tables 1–3, and S1.

#### 3.5.2. Exposure assessment methods for lead

Of the 34 studies that measured lead exposure, nearly all (n = 32) studies measured lead levels in blood. Of these, a few studies concurrently measured lead levels using other sources: 1 study examined urine, hair, and nails [70], 1 examined teeth [58], and 1 examined bone [71]. One study only measured lead levels in dental enamel [57], and another measured lead levels using graphic furnace atopic absorption spectrophotometry [73].

#### 3.5.3. Maternal lead exposure during pregnancy and child neurodevelopment

Three studies reported a negative association prenatal lead levels and at least 1 domain of neurodevelopment [47, 48, 68]. One study reported that lead levels during the third trimester of pregnancy, but not the second, had a negative association with IQ [68], Unexpectedly, low lead levels (geometric mean of 1.75 μg/dL (95% CI: 1.68–1.84)) were associated with a higher BSID score in one study [110].

#### 3.5.4. Postnatal exposure to lead and child neurodevelopment

Twenty-nine studies reported a negative association between lead and at least 1 aspect of neurodevelopment and 3 studies reported no significant effect.

*3*.*5*.*4*.*1*. *Infant development*. Seven studies reported a negative association between lead levels and infant development [48–54], and 2 reported no significant association [56, 101]. One reported no significant association between lead levels and motor function, communication/language, and social adaptation [56], and the other reported no significant association between lead levels and scores within the BSID [101].

*3*.*5*.*4*.*2*. *Academic achievement*. Of the 5 studies that evaluated the relationship between lead exposure and academic achievement, 4 studies reported a negative association [58, 64, 65, 72], and 1 reported no significant difference between lead and academic achievement [55].

*3*.*5*.*4*.*3*. *General intelligence*. Fourteen studies reported a negative association between lead levels and general intelligence [53, 58, 60–71]. One study, however, only reported a negative association between lead exposure and VIQ, but not the Performance Intelligence Quotient (PIQ) or FSIQ [65]. Another reported that lead had no significant association with PIQ [53], and another reported no significant association with any of the subtests of the Wechsler Preschool and Primary Scale of Intelligence (WPPSI) [101].

*3*.*5*.*4*.*4*. *Behavior*. Of the 7 studies that analyzed the effect of lead exposure on behavior, 4 reported a negative association between lead exposure and behavior [55–57, 59]; 1 reported a negative association between behavior and tooth lead levels, but not with blood [58], and 2 studies reported no significant association between lead exposure and behavior [70, 101].

*3*.*5*.*4*.*5*. *Other sub-domains*. Lead was also negatively associated with visuospatial memory [74], the learning of unfamiliar tasks, psychomotor skills [75], receptive vocabulary, visual short-term memory [72], mental maturity, visual spatial abilities [59], and visual-motor maturity [76]. However, lead exposure was not significantly associated with language performance [102], figure matching, figure design, attention, visual selective attention and visual-motor speed, stimulus discrimination [72], numerical reasoning, short-term auditory attention and memory, or general cognition, as measured by the Preuba de Habilidades Cognitivas [75], or Autism Spectrum Disorder [103], and positively associated with the Gross-motor composite subtest from the Bruininks Osteretsky Test of Motor Proficiency and picture vocabulary subtest of the Woodcock Johnson Psycho-Educational battery [101]. Lead was also negatively associated with scores on the Childhood Psychopathological Measurement Schedule [73].

### 3.6. Manganese

#### 3.6.1. Study characteristics

The 11 studies were conducted in 6 different countries, including Bangladesh (n = 1), Brazil (n = 5), China (n = 1), Ecuador (n = 1), Jamaica (n = 1) and Mexico (n = 2). These studies were published between 2010 and 2018 and examined children ranging from birth to 12 years of age. These studies involved a total of 3525 participants ranging from 63 to 1265 participants per study. Eight were cross-sectional studies, 2 were prospective cohort studies that measured manganese levels during at least 2 time points, and 1 was a case control study. Four studies measured and controlled for lead, 1 measured and controlled for arsenic, 6 studies only measured manganese. All studies were rated to have “good” quality. Additional details about these studies can be found in Tables 1–3, and S1.

#### 3.6.2. Exposure assessment methods for manganese

Of the 11 studies that measured manganese exposure, 6 studies measured manganese levels in blood [78, 79, 84–87], 6 measured manganese levels in hair [80–84, 87], 2 measured manganese in water [77, 85], and 1 measured manganese in urine [85].

#### 3.6.3. Maternal manganese exposure during pregnancy and child neurodevelopment

There was only 1 study [77] that measured prenatal manganese levels. This study reported a positive association between maternal water manganese levels during pregnancy and conduct problems. It also reported a negative association between maternal water manganese levels during pregnancy and emotional problems in boys and prosocial scores in girls. Prenatal water manganese levels did not have a significant association with WISC-IV scores.

#### 3.6.4. Postnatal exposure to manganese and child neurodevelopment

All 11 studies that measured postnatal manganese levels found a negative association between manganese levels and at least 1 domain of neurodevelopment.

*3*.*6*.*4*.*1*. *Infant development*. Two studies reported a negative association between manganese levels and neurodevelopment [78, 79]. However, 1 reported no association between blood manganese levels and Psychomotor Developmental Index (PDI) scores on the BSID [78].

*3*.*6*.*4*.*2*. *IQ*. Three studies reported that hair manganese levels were negatively associated with at least 1 domain of general intelligence [82–84]. However, 1 study reported no significant association between blood manganese levels and IQ [84]. There was also no association between hair manganese levels and other subtests of general intelligence such as vocabulary, Corsi blocks forward, Corsi blocks backward, reaction time, trials to complete the first category, and the preservative errors in the Wisconsin Card Sorting Test [83].

*3*.*6*.*4*.*3*. *Behavior*. Three studies reported that manganese was positively associated with poor behavior, including poor attention, an increase in externalizing behavior, hyperactivity, and conduct problems [77, 80, 81].

*3*.*6*.*4*.*4*. *Other domains of neurodevelopment*. In addition to the results listed above, a negative association was reported between hair manganese levels and list memory, immediate recall, learning, and visuospatial working memory [80, 87]. One study reported that blood manganese levels were negatively associated with visual attention, visual perception, and phonological awareness and water manganese levels are negatively associated with language and executive functions [85]. One study, however, reported that although hair manganese levels were negatively associated with blood neurodevelopment, blood manganese levels showed no relationship [87].

*3*.*6*.*4*.*5*. *Autism*. Only 1 study examined the relationship between blood manganese concentrations and autism. No significant association was found between these 2 variables [86].

### 3.7. Mercury

#### 3.7.1. Study characteristics

The 19 studies were conducted in 4 different countries, including Bangladesh (n = 1), Brazil (n = 14), China (n = 3), and Ecuador (n = 1). These studies were published between 1999 and 2021 and examined children ranging from birth to 15 years. Eleven studies were cross-sectional, 6 were prospective cohort studies that measured mercury levels at other time points, 2 were prospective cohort studies that measured mercury levels prenatally. These studies involved a total of 5595 participants that ranged from 70 to 1668 participants per study. None of the studies measured any other heavy metals as confounding variables. All of the studies were rated “good’. Additional details about these studies can be found in Tables 1–3, and S1.

#### 3.7.2. Exposure assessment methods for mercury

Of the 19 studies that measured mercury exposure, 18 measured mercury levels in hair [88–97, 104–109, 112, 113], 4 measured mercury levels in blood [90, 97, 105, 111], 1 measured mercury levels in urine [85].

#### 3.7.3. Maternal mercury exposure during pregnancy and child neurodevelopment

Two studies measured prenatal mercury levels. One showed a positive association between mercury levels and infant development [111], and the other one showed a negative association [88].

#### 3.7.4. Postnatal exposure to mercury and neurodevelopment

*3*.*7*.*4*.*1*.*Overall*. Of the 17 studies that analyzed postnatal mercury levels, 8 reported a negative effect between mercury and at least 1 domain of neurodevelopment [90–97], 4 reported no significant effect [104, 105, 107, 109], and 4 reported a positive association in at least 1 domain [106, 108, 112, 113], and 1 was mixed [89].

*3*.*7*.*4*.*2*. *Infant development*. Eleven studies assessed the association between mercury exposure and infant neurodevelopment. Six of the studies found a negative association [89–94], 3 did not find a statistically significant association [104–106], and 2 studies found positive associations [108, 112]. *GDS*: Three studies reported an inverse association between mercury exposure and GDS scores [89, 92, 93], although 1 study reported this association in only 1 of the 3 towns that was assessed [89]. Two studies reported no significant association between mercury exposure and GDS Score [104, 105], and 1 study reported a significant association between family fish consumption and mercury levels and family fish consumption and development [112]. *BSID*: One study found that mercury is negatively associated with the Mental Developmental Index (MDI) scores from the BSID but not with the PDI [91], and another reported a negative association between neonatal mercury exposure with MDI, but only at 24 months in boys [94]. One study reported no significant association between mercury exposure and BSID scores [108] and 1 reported a positive association [106]. *Neonatal behavioral neurological assessments (NBNA)*: One study reported a negative association between mercury exposure and behavior but only in males [90].

*3*.*7*.*4*.*3*. *Intelligence*. Two studies reported no significant association between mercury exposure and FSIQ scores [107, 113]. One study reported no significant association between mercury exposure and IQ [108]. However, another study reported a negative association between the Copying and Recall subtests of the Stanford-Binet Intelligence Test [95].

*3*.*7*.*4*.*4*. *Behavior*. One study reported that the highest tertile of hair mercury levels was associated with a lower prevalence of hyperactivity and peer relationship problems compared to the lowest tertile (median = 395 μg/kg) [113].

*3*.*7*.*4*.*5*. *Other domains of neurodevelopment*. One study reported no significant association between mercury exposure and any of the subtests in the Lefevre Evolutional Neurological Test, which measures motor coordination and sensory perception [109]. Another study reported that child mercury exposure is negatively associated with the relaxed copying score and block score of the Stanford-Binet Copying Test, both of which assess visuospatial memory, and that maternal mercury exposure was also negatively associated with the relaxed copying score and block score [96]. One study reported a negative association between mercury exposure and subsets of the Santa Ana Form Board, which measures motor coordination and dexterity [95]. Another study reported that children with hair mercury levels > 2 μg/g and blood mercury levels > 5 μg/L had significantly worse visuospatial ability than did children with nontoxic levels of mercury exposure [97].

### 3.8. Mixed metals

#### 3.8.1. Study characteristics

The 22 studies were conducted in 7 different countries, including Bangladesh (n = 9), Brazil (n = 4), China (n = 1), Democratic Republic of the Congo (n = 1), Arab Republic of Egypt (n = 2) Mexico (n = 4) [114–134], and Tanzania (n = 1) [135]. These studies were published between 2001 and 2021 and examined children ranging from birth to 16 years of age. These studies involved a total of 9018 participants ranging from 66 to 1652 participants per study. There were 8 cross-sectional studies, 7 prospective cohort studies, of which 3 measured heavy metal levels prenatally, and 2 case-control studies. Thirteen studies included arsenic as 1 of the metals, 3 included cadmium, 13 included lead, 13 included manganese, and 4 included mercury. Additional details about these studies can be found in S1 Table.

Of the studies that analyzed multiple metals together, the most common combinations were arsenic, manganese, and lead (n = 3) [128, 129, 136] and manganese and arsenic (n = 3) [121, 122, 130].

#### 3.8.2. Metal mixtures and neurodevelopment

Of the 4 studies that examined the interactions between multiple metals, 2 studies found an interaction between lead and manganese, 1 found an interaction between arsenic, lead, and manganese, and one found an interaction between mercury and lead, and mercury and arsenic [135]. One study found that lead was negatively associated with IQ only at high manganese levels [125]. A second study showed that, at 12 months, lead had a more significant effect on the PDI at high manganese levels compared to low manganese levels [117], a third study showed that children with high arsenic, manganese, and lead levels had significantly lower BSID–III scores than those with levels below the median level within the cohort [129], and the fourth showed that the combinations of lead and mercury, and arsenic and mercury potentiate the negative effects on child neurodevelopment [135].

## 4. Discussion

In this systematic review, we review and summarize the current state of environmental epidemiologic research evidence regarding heavy metal exposure and neurodevelopment among children in LMICs. Among the studies reviewed, lead and manganese had the most evidence supporting an association with worse neurodevelopmental outcomes in LMICs postnatally. While few studies were performed of arsenic and cadmium, the existing data show that these metals are associated with worse neurodevelopmental outcomes. Postnatal exposure of mercury was found to be detrimental to neurodevelopment in only half of the studies within the review. One of two studies showed a negative association between prenatal mercury and neurodevelopment; the other saw no effect. Limited-to-no data exist on prenatal exposure to manganese. Throughout this review, most studies that were ranked “fair” and “poor” showed a negative association between heavy metal exposure and various domains of neurodevelopment [55, 59, 60, 63, 69, 71, 74, 76]. Only one of the “fair” and “poor” studies showed no association [55], and one study showed a positive association [111]. Although this weakens the available evidence, there is still an abundance of studies within studies that were rated “good” showing a negative association between heavy metals and neurodevelopment. With these findings, we have summarized knowledge on heavy metal exposures experienced by children in LMICs and adverse neurodevelopmental health consequences, as well as identified gaps that require additional evidence to assist in global policy-making process and health guidelines.

One of the most significant gaps noted was the geographic and economic representation of studies coming from LMICs. Although 135 countries are currently classified as LMICs [32], studies from only 19 of these countries met criteria for inclusion. Furthermore, only 3 studies were conducted in sub-Saharan Africa, even though this region bears the highest number of deaths per capita that are attributable to the environmental exposures [137]. Toxicity from heavy metal exposures have been documented within throughout countries in sub-Saharan Africa, such as Kenya [138], Senegal [139], and Nigeria [140], but data regarding their impacts on child neurodevelopment are unknown. Furthermore, 24 of the included studies were part of 7 groups that used data from overlapping cohorts and only 1 low-income country was represented in the review, indicating that the existing data does not represent the landscape of heavy metal exposures impact on neurodevelopment within LMICs. LMICs experience higher rates of environmental exposures, as well as a myriad of additional risk factors for worse neurodevelopment; thus, it is vital that environmental exposures and their impacts on neurodevelopment are studied within these complex environments where detrimental exposure may have compounding effects.

Similarly, this reviewed found inconsistencies with which potentially confounding variables, such as socioeconomic status, maternal education level, and nutrition, were controlled for within the studies. The detrimental effects of the metals measured were likely potentiated by external factors or interactions with other metals within the environment [141]. Thus, more studies are needed that examine the unique risk factors faced in LMIC settings, in addition to exposure to heavy metals, to determine both risk and protective factors that influence the consequences of these environmental exposures.

Among the metals studied, lead and manganese exposure in childhood were identified as being associated with worse neurodevelopmental outcomes within this review. These findings are consistent with the negative impacts found in resource-rich settings resulting from lead and manganese exposure [26, 142–145]. Our review focused on individual-level biomarker data on exposure, rather than ecological study designs. Specific routes of exposures are not necessarily evident when using biomarker-based exposure assessment, in these studies exposures may have included in utero exposure and inhalation and ingestion during childhood [4]. Additional ecological research is needed to understand the mechanisms of exposure within these settings, to aid in informing policy and programs to reduce exposure.

Studies utilizing hair manganese levels, a measure of relatively chronic exposure, consistently demonstrated adverse effects, whereas studies employing blood manganese levels to assign exposure were inconsistent. The use of a biomarker more reflective of chronic exposure may be a more relevant exposure metric than a single blood manganese level, given the relatively short half-life of manganese in blood and significant day to day variation. Hair may reflect the amount of manganese taken up over time [146] and is therefore a much more accurate biomarker of chronic manganese exposure than blood manganese levels, which vary widely in the short-term [147]. Future studies should consider the optimal method of exposure assessment to characterize meaningful exposure periods of interest.

Unexpectedly, the effects of mercury exposure on neurodevelopment in LMICs differed from what we know from high-income settings. Only 2 studies within our review examined the association of prenatal mercury on neurodevelopment, and postnatal mercury had a detrimental effect on neurodevelopment in only half of the studies. The neurotoxic effects of mercury have been widely documented in high-income settings [18, 148, 149]. Mercury, which exists as elemental, inorganic, and organic forms, enters water bodies through industrial pollution, is methylated by algae and bacteria, and enters fish, which is then eaten by humans [150–152]. Fish consumption is the main source of non-occupational mercury exposure in most settings around the world. However, fish consumption also provides a myriad of key nutrients essential to child neurodevelopment, including iodine, vitamin D, zinc, and highly unsaturated omega-3 and omega-6 fatty acids [153]. Fish is particularly beneficial in low-income communities, as they typically have few other sources of high-quality fats, proteins, and nutrients. Most studies on mercury included within this review focused on populations that consumed high amounts of fish. Since fish consumption is associated with better neurodevelopmental outcomes [154–156], it is possible that the beneficial effects of fish consumption outweigh the detrimental impacts of mercury exposure. However, because of collinearity of mercury exposure and fish consumption, it is difficult to disentangle these positive and negative effects. This is an important consideration when making recommendations on mercury exposure. In high-income countries, advisement has been refined to incorporate promotion of fish with low mercury as optimal choices for children and pregnant women.

Besides mercury, most heavy metals within this review had evidence to support negative associations with postnatal environmental exposure and child neurodevelopment. More equivocal results existed for prenatal exposure to heavy metals, such as arsenic and lead, which were the focus of only a limited number of studies. While the neurotoxicity of lead is clearly established and evidence for arsenic is accumulating, conducting high quality studies in LMICs is needed to better understand the risk in these settings which may represent populations with disparate vulnerabilities and protective factors to inform policy and programs. These exposures are ubiquitous. Arsenic, for example, is abundant in the earth’s crust. Human exposure can therefore occur through many different pathways such as mining, smelting, inhalation, drinking water, eating food, especially rice [10, 157]. These potential exposures can be found around the world, 5 of the 6 total prenatal arsenic studies were all conducted in Bangladesh, thus our findings may not be representative of arsenic’s effects worldwide. Even in high-income settings, the evidence has been mixed on whether prenatal arsenic exposure has an effect on neurodevelopment [27, 28]. Prenatal exposure to lead, however, has been shown to have detrimental effects on neurodevelopment in high income countries [152, 158]. Due to the fetal brain’s susceptibility to environmental toxicant, it is critically important that we have clear evidence to guide policy and programs around prenatal arsenic and lead exposure. More research is needed in this area, in both high-income countries and LMICs to inform policy and practice related to prenatal care.

In this review, only 4 studies examined the interactions between multiple metals. Two showed that manganese levels potentiate lead toxicity [80, 117], 1 showed that children with higher levels of arsenic, manganese, and lead had lower cognitive development compared to those with median levels [129], and another showed that arsenic and lead individually potentiate the negative effects on mercury on neurodevelopment [135]. Few studies in high-income settings examine the synergistic effects of multiple metals on neurodevelopment; these studies suggest presence of other heavy metals may increase the neurotoxicity of lead [141]. A study in Korea, for example, showed that lead toxicity increased in the presence of high cadmium levels [159]. In another study in Taiwan, children with high manganese and lead levels had significantly lower developmental scores than those with only high lead levels [22]. Mixtures-based analytical methods are advancing in environmental epidemiology and will be important in future studies to determine the impact of mixed exposures on child neurodevelopment in both high-income countries and LMICs [160].

There are several limitations to this review. First, it is possible that studies which fail to show significant findings related to heavy metal exposure and neurodevelopment may be less likely to be published. Thus, our review may be subject to publication bias. Second, despite the large number of studies included in this review, we were unable to perform a meta-analysis due to the heterogeneity in sampling times, methods of measuring exposure, ages at testing times, diverse settings, and the various neurodevelopment assessment scales that were used. Therefore, it was not possible to quantify how heavy metals affect neurodevelopment in LMICs based on the existing state of the evidence base. Future studies would benefit from harmonized data collection methods in order to pool data together to answer questions about impact of heavy metals on neurodevelopment. And finally, because many of these clinical studies looked at correlations between heavy metals and neurodevelopment, causation cannot be assumed. This is a limitation to all epidemiological studies.

## 5. Conclusions

With this systematic review, we summarized the existing epidemiologic literature on heavy metal exposure and neurodevelopment in LMICs. While we found consistent evidence that postnatal exposure to lead and manganese is associated with worse neurodevelopmental outcomes, other heavy metal exposures had mixed or limited evidence supporting a clear association with worse outcomes in LMICs. The limited scope of the current research base in low-income countries in particular, and the small number of studies evaluating exposure during the perinatal period and early childhood underscores the need for more emphasis on pediatric environmental epidemiologic research in LMICs. This is a critical step to inform intervention approaches, understand potentially protective factors, and create programs and policies to reduce harmful environmental exposures for children living in LMICs.

## Supporting information

S1 Checklist (DOCX)

S1 File Search strategy. (DOCX)

S1 Table Summary of the studies included in the systematic review. (DOCX)
