# Supplementary material for: Heavy metals and neurodevelopment of children in low and middle-income countries: A systematic review
Source: PLoS One. 2022 Mar 31;17(3):e0265536. doi: 10.1371/journal.pone.0265536 (PMC8970501; doi:10.1371/journal.pone.0265536)
Supplement: S1 File — (DOCX) [file pone.0265536.s002.docx]

**S1:** Search Strategy used in PubMed

Search 1*: (((((((((((((((((("Africa"[Mesh]) OR "Developing Countries"[Mesh]) OR "Poverty"[Mesh]) OR "Rural Population"[Mesh]) OR "South America"[Mesh]) OR (africa)) OR (third world)) OR (south america)) OR (rural)) OR (resource limitation)) OR (resource limit)) OR (resource-limited)) OR (resource constraint)) OR (resource-constrained)) OR (poverty)) OR (low-resource)) OR (low-income)) OR (developing countries)) OR (developing country)

Search 2: ((((((((((((((((((((((((((((((("Air Pollution"[Mesh]) OR "Water Pollution"[Mesh]) OR "Environmental Pollution"[Mesh]) OR "Arsenic"[Mesh]) OR "Environmental Exposure"[Mesh]) OR "Metals, Heavy"[Mesh]) OR "Inhalation Exposure"[Mesh]) OR "Iron"[Mesh]) OR "Lead"[Mesh]) OR "Manganese"[Mesh]) OR "Mercury"[Mesh]) OR (polluted environment)) OR (polluted air)) OR (polluted water)) OR (water pollutant)) OR (water pollution)) OR (polluted)) OR (pollutant)) OR (pollution)) OR (mercury)) OR (manganese)) OR (lead)) OR (iron)) OR (inhalation exposure)) OR (heavy metal)) OR (environmental pollutant)) OR (environmental pollution)) OR (environmental exposure)) OR (chemical)) OR (arsenic)) OR (air pollutant)) OR (air pollution)

Search 3: (((((((((((((((((((((((((((((("Attention"[Mesh]) OR "Behavior"[Mesh]) OR "Child Behavior Disorders"[Mesh]) OR ( "Child Development"[Mesh] OR "Developmental Disabilities"[Mesh] )) OR "Cognition"[Mesh]) OR ( "Executive Function"[Mesh] OR "Neuropsychological Tests"[Mesh] )) OR "Hyperkinesis"[Mesh]) OR "Memory, Long-Term"[Mesh]) OR "Memory, Short-Term"[Mesh]) OR "Risk Assessment"[Mesh]) OR "Verbal Learning"[Mesh]) OR (verbal learning)) OR (risk assessment)) OR (neuropsychological test)) OR (neurodevelopmental)) OR (neurodevelopment)) OR (short term memory)) OR (long term memory)) OR (IQ)) OR (intelligence)) OR (hyperkinesis)) OR (executive functioning)) OR (executive function)) OR (developmental disabilities)) OR (developmental disability)) OR (cognitive)) OR (cognition)) OR (child behavioral disorder)) OR (child development)) OR (behavior)) OR (attention)

Search 4: ((((child) OR (children)) OR (childhood)) OR (adolescent)) OR (("Child"[Mesh]) OR "Adolescent"[Mesh])

Search 5: #1 AND #2 AND #3 AND #4

*: Not all locations were represented within this thread. Upon consultation with a medical librarian and review of the embedded keywords and MeSH terms for known relevant articles, this was the recommended string to use to identify studies from low- and middle- income countries.

Full Study Eligibility Criteria

Inclusion

Population: children below the age of 18 including conception living in low-and middle income counties, as determined by World Bank Criteria

Intervention: measurable heavy metal exposure, including the following: actinium, americium, antimony, barium, berkelium, bismuth cadmium, californium, cesium, cesium isotopes, chromium, chromium isotopes, cobalt, copper, curium, einsteinium, fermium, francium, gallium, gallium isotopes, germanium, gold, gold isotopes, hafnium, indium, iridium, iron, iron isotopes, lawrencium, lead, manganese, mendelevium, mercury, mercury isotopes, molybdenum, neptunium, nickel, niobium, nobelium, osmium, palladium, platinum ,plutonium, protactinium, radium, rhenium, rhodium, rubidium, ruthenium, silver

Outcome: standardized measurement of cognitive or behavioral functioning

Measurable outcomes (systematic procedure for measuring outcomes)

Language: English Only

Study Design: observational cohorts, cross-sectional, quasi experimental, ecological

Full-text articles only

Peer reviewed articles and published theses are included

Exclusion

Reviews and editorials (only original studies will be included)

Abstracts only

Studies that do not measure or document potential confounding factors related to cognition

Studies conducted before 1970

Case studies

Studies without cognitive development data for each subject (collective population data does not suffice)

Study type is NOT one of the following: observational cohorts, cross-sectional, quasi experimental, ecological

Studies in any language other than English
