# Supplementary material for: Heavy metals and neurodevelopment of children in low and middle-income countries: A systematic review
Source: PLoS One. 2022 Mar 31;17(3):e0265536. doi: 10.1371/journal.pone.0265536 (PMC8970501; doi:10.1371/journal.pone.0265536)
Supplement: S1 Table — (DOCX) [file pone.0265536.s003.docx]

**S1 Table. Summary of the studies involving arsenic included in the systematic review.**

| Author | Study design | Country | Population | Age at outcome measurement | Prenatal measurement | Method of measuring metal exposure | Neurological assessment | Concentration | Results | Covariables adjusted for within the analyses | Quality assessment |
| --- | --- | --- | --- | --- | --- | --- | --- | --- | --- | --- | --- |
| Asadullah and Chaudhury, (2011) [1] | Cross-sectional | Bangladesh | n = 7479 | 13 years | No | Water: asked children whether their tube well was painted red (> 50 µg/L) | Two math tests that were administered to all students | 88.5% < 50 µg/L 11.5% > 50 µg/L | Negative correlation between arsenic exposure and children’s math scores, ranging from -0.08 to -0.17 standard deviations for the primary-standard mathematics test and -0.05 and -0.07 standard deviations for the secondary-standard test (p < 0.05) | Sociodemographics, water manganese | Good |
| Hamadani, et al. (2010) [2] | Cohort | Bangladesh | n = 2112 | 18 months | Yes | Urine | Wolke's Behavior Rating Scale, Bayley Scales of Infant Development-II, language development based on mothers' report | Median (IQR^[[1]](#footnote-1)^), calculated from both measurements Mother's urine from gestational week (GW^[[2]](#footnote-2)^) 9 and 30: 96.3 (46, 219) µg/L Child's urine: 34.6 (18, 80.2) | No association between arsenic levels at any time point and any of the tests | Age, gestational age, HOME^[[3]](#footnote-3)^ score, weight-for-height z score, mother’s education, assets | Good |
| Hamadani, et al. (2011) [3] | Cohort | Bangladesh | n = 2853 | 5 years | Yes | Urine | Wechsler Preschool and Primary Scale of Intelligence | Median (10th and 90th percentiles) GW 8: 81 (24, 380) µg/l GW 30: 84 (26, 415) µg/l 1.5 years: 34 (12, 155) µg/l 5 years: 51 (20, 238) µg/l | Maternal arsenic levels in urine during pregnancy was negatively associated with Verbal Intelligence Quotient (VIQ^[[4]](#footnote-4)^) (β = -1.52; 95% confidence interval (CI^[[5]](#footnote-5)^): -2.6 to -0.4) and Full Scale Intelligence Quotient (FSIQ^[[6]](#footnote-6)^) (β = -1.35; 95% CI: -2.4 to -0.3) for girls only  Concurrent arsenic levels in urine at the time of showed a negative association in girls for VIQ (β = 02.4, 95%CI: -3.8 to -1.1) and FSIQ (β = -1.4; 95%CI: -2.7 to -0.1) | Socioeconomic status, housing, assets, income-expenditure deficits, maternal BMI at enrolment, maternal age, parity, parents’ education, gestational age, birthweight, length and head circumference | Good |
| Rocha-Amador, et al. (2007) [4] | Cross-sectional | Mexico | n = 132 | 6-10 years | No | Arsenic: water Lead: blood | Wechsler intelligence for Children, Revised Mexican edition (WISC-RM^[[7]](#footnote-7)^) | Mean (standard deviation (SD^[[8]](#footnote-8)^)): Arsenic:  Moctezuma: 12.6 (2.0) µg/g crt Salitral: 116 (2.2) µg/g crt Cinco de Febrero: 52.5 (2.2) µg/g crt  Lead Moctezuma: 7.1 (2.2) µg/dL Salitral: 6.2 (2.1) µg/dL 5 de Febrero: 4.8 (3.4) µg/dL | Water arsenic was negatively associated with VIQ (β = -6.40), Performance Intelligence Quotient (β = -4.30) and FSIQ (β = -6.14), p < 0.001 | Blood lead, mother’s education, socioeconomic status, height-for-age z score, transferrin saturation | Good |
| Rosado, et al. (2007) [5] | Cross-sectional | Mexico | n = 557 | 6-8 years | No | Arsenic: urine Lead: blood | WISC-RM subscales: Coding, Digit Span, Arithmetic Number and Letter sequencing Cognitive Abilities test Math Achievement test Visual-Spatial Abilities with Figure Design Peabody Picture Vocabulary Test Sternberg Memory Visual Memory Span Visual Search | Mean (SD) Arsenic:  58.1 (33.2) µg/L  Lead 11.5 (6.3) µg/dL | Arsenic negatively associated with Visual-Spatial abilities with Figure Design (β = -0.024, 95% CI: -0.045, -0.004; p < 0.05), Peabody Picture Vocabulary Test (β = -0.064, 95% CI: -0.115, -0.013; p < 0.05), WISC-RM Digit Span Subtest (β = -0.014, 95% CI: -0.025, -0.002; p < 0.05), Visual search (β = -0.007, 95% CI: -0.011, -0.002; p < 0.01), and letter sequencing (correct trials 0 vs ≥ 1)(β = -0.992, 95% CI: -0.987, -0.996 p < 0.01)  No significant correlation with math achievement, WISC-RM Arithmetic Subtest, Sternberg Memory, Visual Memory Span, WISC-RM coding subtest, and Stimulus Discrimination | Hemoglobin concentration, blood lead, sociodemographic confounders | Good |
| Roy, et al. (2011) [6] | Cross-sectional | Mexico | n = 527 | 6-7 years | No | Urinary arsenic, blood lead | Connors Behavior Rating Scales | Median (IQR) Arsenic: 55.2 (39.7) µg/L Lead: 10.0 (6.6) µg/L | There was no significant difference between arsenic levels and behavior | Age, sex, maternal education, family socio-economic status, hemoglobin, blood lead levels, mother’s education | Good |
| Siripitayakunkit, et al. (1999) [7] | Cross-sectional | Thailand | n = 529, total sample n = 353 living in the Ronpiboon subdistrict n = 176 living in the Soa Thong subdistrict | 6-9 years | No | Hair | Wechsler Intelligence Scale for Children (WISC^[[9]](#footnote-9)^) | Mean (SD) 3.52 (3.58) µg/g | Arsenic explained 14% of the variance in IQ scores (p = 0.002) | Father’s occupation, maternal intelligence score, family income | Good |
| Tofail, et al. (2009) [8] | Cohort | Bangladesh | n = 1799 | 7 months | Yes | Urine | Psychomotor Developmental Index from Bayley Scales of Infant Development-II Support and Cover: two one-step means-end problem solving tests A modified version of Wolke's Behavior Rating Scale | Median (IQR) GW 8: 81 (37-207) µg/L GW 30: 84 (42-230) µg/L | No significant correlation between mother's urinary arsenic and any of the tests | Assets, father’s educational level, mother’s BMI, child’s gestational age, birth length, current high, sex, and age | Good |
| Vahter, et al. (2020) [9] | Cohort | Bangladesh | n = 1523 | 10 years | Yes | Urine, hair. blood | WISC-IV | Median (range) of the third quintile Maternal urinary arsenic, GW8: 68 (7.4 - 1122) µg/L Maternal erythrocyte arsenic, GW14: 4.1 (0.57- 56.6) µg/L Urinary arsenic at 5 years: 47 (12-813) µg/L Urinary arsenic at 10 years: 57.3 (45.6 - 73.1) Hair arsenic at 10 years: 396 (99-3297) ng/g Water arsenic at 10 years: 1.58 (0.01-510) µg/L Hair selenium at 10 years: 489 (83.7) ng/g Urinary cadmium at 10 years: 0.25 (0.04-2.5) µg/L Urinary lead at 10 years: 1.5 (0.16-16.0) µg/L Water manganese at 1 years: 419 (0.5-7266) µg/L | Children with maternal urinary arsenic levels between 122 and 246 µg/L at GW 8 had lower FSIQ scores compared than the lowest quintile (3.2 - 32.4) µg/L (β: -5.98; 95% CI: -10.2 to -1.77). However, there was no significant difference in those with urinary arsenic levels > 247 µg/L  There was no significant association between maternal urinary arsenic concentrations at GW 30 and development   Children's urinary arsenic levels at 5 years had no significant association with FSIQ  At 10 years, children with urinary arsenic levels between 73.4 and 162 µg/L had lower FSIQ than children with levels < 30 µg/L (β: -6.37; 95% CI: -10.5 to -2.22). However, there was no significant difference in IQ score in children with arsenic levels > 163 µg/L | Parental education, maternal age, body weight, height, and smoking, socioeconomic status | Good |
| Von Ehrenstein, et al. (2007) [10] | Cohort | India | n = 351 | 5-15 years | Yes | Urine, water | WISC:  Verbal subtests: Vocabulary, Digit Span Performance-type subtests: Object Assembly, Coding, Picture Completion, Block Design  Raven’s Colored Progressive Matrices RCPM Total Sentence Recall Test A version of the Purdue pegboard test | Mean (SD) Water in pregnancy: 110 (243) µg/L Child urine: 78 (61) µg/L | Compared to children with < 43 µg/dL of urinary arsenic, those with urinary arsenic levels > 82.6 µg/dL had a decline of 12% (95% CI: 0.4-24%) on the vocabulary test, 21% (95% CI: -0.8% to 42%) on the object assembly test, and 13% (95% CI: 0.3% to 24%) in the picture completion test  There was no significant association between test results and water arsenic concentrations during pregnancy or childhood | Age, sex, BMI, maternal and paternal education, father’s occupation, mother’s age, type of house building material, number of rooms in the house | Good |
| Wang, et al. (2007) [11] | Cross-sectional | China | n =720 living in rural areas in Shanxi, in central Northern China n = 196: control n = 253: high fluoride n = 91: medium arsenic n = 180: high arsenic | 8-12 years | No | Water, urine | Combined Raven's Test- the Rural | Mean (SD) Low level group:  Water: 2 (3) µg/L Urine: 10 (2) µg/L Medium level group:  Water:142 (106) µg/L Urine: 46 (3) µg/L  High level group:  Water: 190 (183) µg/L Urine: 73 (3) µg/L | Water arsenic levels negatively correlated with IQ scores:  Mean IQ scores decreased from 105±15 in the control group (2±3 µg/L) to 101±16 in the medium-As group 142±106 µg/L) (p < 0.05) to 95 ±17 in the high arsenic group (190±182 µg/L) (p < 0.01) | No covariates used | Good |

Table B: Summary of the studies involving cadmium included in this systematic review

| Author | Study design | Country | Population | Age at outcome measurement | Prenatal measurement | Method of measuring metal exposure | Neurological assessment | Concentration | Results | Covariables adjusted for within the analyses | Quality assessment |
| --- | --- | --- | --- | --- | --- | --- | --- | --- | --- | --- | --- |
| Gustin, et al. (2018) [12] | Cohort | Bangladesh | n = 1453 | 10 years | Yes | Urine | Wechsler’s Intelligence Scale for Children - IV and Strengths and Difficulties Questionnaire | Median (5th - 9th percentile) Mothers during early pregnancy: 0.63 (0.18-2.0) µg/L 5 years of age: 0.22 (0.083- 0.66) µg/L 10 years of age: 0.24 (0.083- 0.64) µg/L | Cadmium is negatively associated with the Full Scale Intelligence Quotient, as children in the highest tertile of urinary cadmium (range: 0.30 -2.6 µg/L) at 10 years scored 4.9 points lower (95% confidence interval (CI^[[10]](#footnote-10)^): -8.1, -1.7; p = 0.002) than the children in the lowest tertile (0.036-0.18 µg/L)  Urinary cadmium is negatively associated with prosocial behavior (Odds Ratio: 0.48; 95% CI: 0.25 to 0.93; p for trend = 0.022) and positively associated with hyperactivity in girls only (OR: 2.2; 95% CI: 1.2 to 3.9, p for trend = 0.011) | Maternal age, weight, height, parental education, smoking habits during pregnancy, gestational age. 5 year follow up: maternal IQ. 10 year follow up: number of children in the household, years of childrens’ formal schooling, type of school attended, HOME score, height and weight, hemoglobin concentration, children’s socioeconomic status. | Good |
| Tian, et al. (2009) [13] | Cohort | China | n = 106 | 4.5 years | Yes | Blood | Wechsler Preschool and Primary Scale of Intelligence, Revised | Median (range) Cadmium Maternal blood: 1.8 (0.43- 25.25) µg/L Cord blood: 0.60 (0.02- 1.78) µg/L Placenta: 0.15 (0.03-3.97) µg/g dry weight  Lead Maternal blood: 72.4 (23.1- 186. 5) µg/L Cord blood: 43.6 (17.2 - 98.2) µg/L Placenta: 0.6 (0.5-1.35) µg/L | Performance Intelligence Quotient was significantly lower in children with cord cadmium ≥6 µg/L compared to those with <0.6 µg/L (100±12 vs 95±10; p < 0.05)  Full Scale Intelligence Quotient was significantly lower in children with cord cadmium ≥6 µg/L compared to those with <0.6 µg/L (99±8 vs 95 ±6; p < 0.05)  No correlation between cadmium and Verbal Intelligence Quotient | Cord blood lead, maternal age, height, weight, gestational weeks, maternal educational status, method of delivery, feeding methods, nursery school age, environmental tobacco smoke, per capita annual income of the family | Good |
| Wang, et al. (2016) [14] | Cohort | China | n = 149 | 12 months | Yes | Blood | Gesell Developmental Scale | Mean (range):  Maternal blood: 1.18 (0.13-4.55) µg/L | A 10-fold increase in the maternal cadmium levels was associated with a 5.70 point decrease (95% CI: -10.91 to -0.49; p = 0.032) in the social domain No significant association in any of the other subtests | Maternal age, maternal IQ, maternal education, smoking during pregnancy, meat consumption, paternal education, household monthly income, infant gender, birth weight, gestational age, maternal blood lead and maternal blood mercury | Good |

Table C: Summary of the studies involving lead included in this systematic review

| Author | Study design | Country | Population | Age at outcome measurement | Prenatal measurement | Method of measuring metal exposure | Neurological assessment | Concentration | Results | Covariates adjusted for within the analyses | Quality assessment |
| --- | --- | --- | --- | --- | --- | --- | --- | --- | --- | --- | --- |
| AbuShady, et al. (2017) [15] | Cross-sectional | Egypt | n = 400, total population n = 200, living in Helwan, an industrial area n = 200, living in Dokki, an urban area | 6-12 years | No | Blood | Teachers and other school staff were asked to assess each child's performance and behaviors" | Mean (standard deviation (SD^[[11]](#footnote-11)^)) industrial: 10.37 (7.94) µg/dL Urban: 5.45 (3.9) µg/dL | Blood lead levels ≥10 µg/dL positively associated with abnormal behavior (p < 0.05) but not with school achievement | Residence, health habits, housing exposure, age, sex | Fair |
| Alvarez-Ortega, et al. (2017) [16] | Cross-sectional | Colombia | n = 118 | 5-16 years | No | Blood | Kaufman Brief Intelligence test (Vocabulary and matrices) | Mean (SD): 1.7 (0.3) µg/dL | Blood lead levels had a negative correlation with total score (ρ = −0.401, P < 0.001), verbal (ρ = −0.405, P < 0.001), matrices (ρ = −0.317, P < 0.001) subtests | Age, weight, height, BMI, hematologic parameters, biochemical parameters | Fair |
| Bellinger, et al. (2005) [17] | Cross-sectional | India | n = 74 | 4-14 years | No | Blood | Binet-Kamath Intelligence Test, Wide Range Assessment of Visual Motor Abilities, Connors Behavioral Rating Scale | Mean (SD) 11.1 (5.6) µg/dL | There is a negative association between blood lead levels and IQ levels, as children in the highest and lowest blood lead quartiles had mean IQs of 95.6 ± 13.3 and 102.0 ± 22.5, respectively | Maternal age, maternal education, average monthly income of family, sex, age, grade, early feeding method | Good |
| Braun, et al. (2012) [18] | Cross-sectional | Mexico | n = 1035 | 4 years | No | Blood | McCarthy Scales of Children's Abilities | Median (range) 1 year: 4.2 (0.3-27.4) µg/dL 2 years: 4.6 (0.6-36.8) µg/dL 3 years: 5.5 (0.6-50.1) µg/dL 4 years: 5.9 (0.3-30.3) µg/dL | 2-year blood lead concentrations are most strongly associated with reduced developmental scores (β = -7.1; 95% confidence interval (CI^[[12]](#footnote-12)^): -12, -2) | Maternal education, IQ, marital status, duration of breastfeeding, child sex, study cohort | Good |
| Counter, et al. (1998) [19] | Cross-sectional | Ecuador | n = 77 | 4-15 years | No | Blood | Group 1: Evaluation of cranial nerve function, gross motor skills, fine motor skills, and deep tendon reflexes, right/left discrimination, size discrimination, color discrimination, draw-a-person, and simple math calculation  Group 2: Raven’s Colored Progressive Matrices (RCPM^[[13]](#footnote-13)^) | Mean (SD) Group 1, the neurological test group: 48.0 (26.4) µg/dL Group 2: the test group administered the RCPM: 47.4 (22.0) | There is an inverse correlation between blood lead levels and RCPM scores for children ages 9 and older (r = -0.618; p = 0.011) There was a significantly higher blood lead level in children with abnormal vs normal RCPM scores (54.3± 20.4 vs 39.5± 21.4, respectively, p = 0.030) | None found | Good |
| Counter, et al. (2005) [20] | Cross-sectional | Ecuador | n = 188 | 5-11 years | No | Blood | RCPM | Mean (range) 29.3 (3.5-94.3) µg/dL | There is an inverse correlation between blood lead levels and RCPM standard score (r = -0.331, β = -0.189, p= 0.0001). | None found | Fair |
| Counter, et al. (2008) [21] | Cross-sectional | Ecuador | n = 166 | 6-16 years | No | Blood | Digit Span subtest of the Wechsler Intelligence Scale for Children (WISC^[[14]](#footnote-14)^) -IV | Mean (SD) Lead: 18.0 (15.1) µg/dL Zinc protoporphyrin/heme: 105.7 (100.9) umol/mol | There is an inverse correlation between digit span score and blood lead level (r= 0.251, p = 0.001) | None found | Fair |
| do Nascimento, et al. (2014) [22] | Cross-sectional | Brazil | n = 40, total population n = 20, living in a rural area in the central region of Rio Grande do Sul, Southern Brazil n = 20, living in a urban area in the central region of Rio Grande do Sul, Southern Brazil | 8-14 years | No | Blood (arsenic, cadmium, nickel, lead, cobalt, copper, manganese, selenium)  hair (aluminum, arsenic, cadmium, nickel, lead, cobalt, copper, selenium)  drinking water (aluminum, arsenic, cadmium, mercury, nickel, lead) | Bender test for visual motor skills. R-2 intelligence test | Mean (SD), rural; urban not measured  Blood Arsenic: 3.95 (0.08) µg/L Cadmium: 0.04 (0.01) µg/L Lead: 42.06 (8.71) µg/dL Nickel: 4.64 (1.39) µg/L Cobalt: 0.19 (0.02) µg/L Copper: 102.7 (25.56) µg/L Manganese: 11.21 (0.53) µg/L Selenium: 72.33 (2.342) µg/L  Hair Aluminum: 52.0 (9.0) µg/g Arsenic: 0.04 (0.008) µg/g Cadmium: 0.28 (0.09) µg/g Mercury:: 0.19 (0.03) µg/g Lead: 1.46 (0.27) µg/g Nickel: 0.08 (0.008) µg/g Cobalt: 0.06 (0.01) µg/g Copper: 12.88 (2.50) µg/g Selenium: 0.65 (0.05) µg/g  Drinking water Aluminum: 0.04 (0.09) mg/L Arsenic: 0.001 mg/L Cadmium: 0.001 mg/L Mercury: 0.002 (0.0002) mg/L Nickel: 0.002 (0.0007) mg/L Lead: 0.002 (0.0002) mg/L | Lead levels significantly associated with visual-motor immaturity (p < 0.01) | None found | Poor |
| Gahyva, et al. (2008) [23] | Cross-sectional | Brazil | n = 20 | 3-6 years | No | Blood | Phonology Task from the ABFW-Child Language Test; Vocabulary task from the ABFW-Child Language test; Peabody Picture Vocabulary Tests, Morphosyntax Evaluation Protocol, Pragmatic Skills Evaluation Protocol, Bucofacial and Articulation Praxis Protocol, Hearing Immediate Memory, through the Hearing sequential memory fo the Illinois Test of Psycholinguistic Abilities | Range:  10.5-35.8 µg/dL | No significant association between blood lead levels and language performance | None found | Good |
| Gleason, et al. (2020) [24] | Cohort | Bangladesh | n = 734 | 20 – 40 months | No | Blood | BSID- III | Mean (SD)  Venous blood Arsenic: 1.0 (0.8) µg/dL  Venous blood manganese: 2.1 (2.1) µg/dL  Venous blood lead: 4.5 (2.1) µg/dL | In areas of low blood lead, a 1-unit increase in natural log cord blood lead µg/dL in the presence of stunting was associated with a 2.10 decrease in cognitive scores (p= 0.003) | Stunting, maternal height and weight, maternal education, maternal protein intake, HOME score^[[15]](#footnote-15)^, maternal IQ | Good |
| Kamel, et al. (2003) [25] | Cross-sectional | Egypt | n = 250 | 10-12 years | No | Blood | IQ test (not specified) School grades at the end of the year | Mean (SD):  17.36 (10.67) µg/dL | Blood lead levels negatively associated with IQ scores (r = -0.8150, p < 0.001), Arabic test scores (r = -0.7063, p < 0.001), mathematics test scores (r = -0.8022; p < 0.001), and final test scores (r = -0.8891, p < 0.001) | Age, crowding index, number of missed school days | Good |
| Kashala-Abotnes, et al. (2016) [26] | Cross-sectional | Democratic Republic of the Congo | n = 89 | 12-24 months | No | Blood | The Gensini-Gavito Scale and the Baby Characteristics Questionnaire | Mean (SD) 6.9 (4.8) µg/dL | Blood lead levels not significantly associated with development  More children with levels ≥ 5 µg/dL perceived as difficult compared to those with levels between 0 and 4 µg/dL (p = 0.002) Significant higher proportion of children reported with difficult temperament among those with levels ≥ 10 µg/dL (22/23) and at 5- 9 µg/dL (22/36) compared to those at 0-4 µg/dL (15/30) (p = 0.002) Children with blood lead levels at 5-9 and ≥ 10 µg/dL: higher mean rank on the Baby Characteristics Questionnaire (p < 0.05) | Sociodemographic factors, mother and child history around pregnancy and birth, overall environmental exposure to lead | Good |
| Kordas, et al. (2004) [27] | Cross-sectional | Mexico | n = 602 | 6-8 years | No | Blood | Cognitive Abilities Test; Wechsler Intelligence Scale for Children, Revised Mexican Version (WISC-RM^[[16]](#footnote-16)^) coding, digit retention and arithmetic; number and letter sequencing; Prueba de Habilidades Cognitivas (a computer-based test of cognitive abilities with 5 subtests, a curriculum-based Math achievement test, a test of visual-spatial abilities and the Peabody Picture Vocabulary est | Mean (SD) 11.5 (6.1) µg/dL | Compared to children with blood lead levels < 10 µg/dL, children with level ≥20 µg/dL have a significantly lower score on the Peabody Picture Vocabulary Test (106.3 ± 14.6 vs 95.3 ± 15.0), WISC-RM Coding (11.4 ± 2.7 vs 10.4 ± 2.7), Number Sequencing (6.3 ± 3.0 vs 5.2 ± 2.5) and letter sequencing tests (54% vs 33%); p< 0.05. There was no significant difference between blood lead levels and the WISC-RM arithmetic, WISC-RM digit span, or Preuba de Habilidades Cognitivas score | Age, gender, socioeconomic status, school | Good |
| Kordas, et al. (2006) [28] | Cross-sectional | Mexico | n = 602 | 6-8 years | No | Lead: blood Arsenic: urine | Math achievement, Peabody picture Vocabulary Test, Freedom from Distractibility Factor, Sequencing, Sternberg Memory, Figure Matching, Figure Design, Visual Search | Mean (range) Lead:  10.2 (2-43.8) µg/dL Arsenic: Not published | Blood lead levels negatively correlated with math achievement test (β = -0.17, 95% CI: -0.28; -0.06; adjusted R^2^ = 23.4; p < 0.001) Peabody picture vocabulary test (β = -0.36, 95% CI: -0.58; -0.13; adjusted R^2^ = 23.6; p < 0.001), Sternberg memory test (β = -0.05, 95% CI: -0.10; -0.01; adjusted R^2^ = 13.4; p < 0.05). blood lead levels not significantly correlated with the freedom from distractibility factor, sequencing, figure matching, figure design, visual search, and the number of errors in the cognitive abilities test | Age, gender, family socio-economic status, maternal formal education, parental involvement in schooling, family structure, birth order | Good |
| Kordas, et al. (2011) [29] | Cohort | Mexico | n = 186 | 24 months: Bayley Scales of Infant Development (BSID^[[17]](#footnote-17)^)-II 48 months: McCarthy Scales of Infant Development | No | Blood | BSID-II McCarthy Scales of Infant Development | Mean (SD) Cord blood: 6.6 (3.3) µg/dL 24 months: 8.1 (4.4) µg/dL 48 months: 8.1 (3.6) µg/dL | Cord lead levels negatively associated with Mental Developmental Index (MDI^[[18]](#footnote-18)^) (p < 0.01) and Psychomotor Developmental Index (PDI^[[19]](#footnote-19)^) (p < 0.1) but not McCarthy scores  24-month blood lead levels not significantly associated with any score 48-month blood lead levels: inversely associated with McCarthy general cognitive index (GCI^[[20]](#footnote-20)^) (p < 0.05) and memory scores (p < 0.1) | Birth weight, gestational age, child sex, maternal age, maternal years of schooling, maternal IQ, maternal marital status, crowding in the house, type of floor in the house | Good |
| L, et al (2021)  [30] | Cross-sectional | India | n = 72 | 9-15 years | No | Graphite furnace atomic absorption spectrophotometry | Childhood Psychopathological Measurement Schedule | Mean (IQR): 4.95 µg/dL (4.47) | Lead was negatively associated with intelligence (p = 0.002), and positively associated with anxiety (p = 0.0002), depression (p <0.0001), psychotic symptoms (p = 0.0001), and somatization (p = 0.04) | None found | Good |
| Liu, et al. (2013) [31] | Cohort | China | n = 561 | 6 years: Wechsler Preschool and Primary Scale of Intelligence (WPPSI^[[21]](#footnote-21)^) 8-10 years: standardized tests in Math, English, and Chinese | No | Blood | WPPSI, standardized tests in Math, English, and Chinese | Mean (SD) 6.43 (2.64) µg/dL | Blood lead levels negatively associated with Chinese (r = -0.234, p < 0.001), Math (r = -0.200, p < 0.001), and English (r = -0.207, p < 0.001) Blood lead levels negatively associated with the Performance Intelligence Quotient (PIQ^[[22]](#footnote-22)^) (r = -0.056, p < 0.05) but not the Verbal Intelligence Quotient (VIQ^[[23]](#footnote-23)^) or Full Scale Intelligence Quotient (FSIQ^[[24]](#footnote-24)^) | Age, gender, residence, blood iron level, parent education, parent occupation, father’s smoking | Good |
| Mostafa, et al. (2009) [32] | Cross-sectional | Egypt | n = 100 | 6- 12 years | No | Blood | WISC-III | Median:  Total: 9 µg/dL High lead population: 11.5 µg/dL Low lead population: 6 µg/dL | Children with blood lead levels ≥10 µg/dL had higher cognitive dysfunction (81.4%) than those with levels < 10 µg/dL (3.5%; p < 0.001); children with cognitive dysfunction had higher blood lead levels (median 14 µg/dL) than those with normal cognitive function (median 7 µg/dL; p < 0.001) | None found | Good |
| Olympio, et al. (2010) [33] | Cross-sectional | Brazil | n = 173 | 14-18 years | No | Dental enamel | Self-reported delinquency form, Child Behavior Checklist | Mean (SD) 12.4 (16.0) µg/g | There was a significant difference in somatic complaints (p = 0.01), somatic problems (p =0.01), social problems (0.04) and externalizing behavior (p = 0.02) between high and low level groups  Dental enamel concentration positively associated with somatic complaints (OR: 2.93; 95%CI: 1.09, 7.91), social problems (OR: 3.04; 95%CI: 1.07, 8.64), rule-breaking behavior (OR: 3.72; 95%CI: 0.99, 14.04), and externalizing behavior (OR: 2.87; 95%CI: 1.05, 7.85)  There was not a significant difference in withdrawn behavior (p = 0.63), anxious/depressed mood (p = 0.99), social competencies (p = 0.19), thought problems (p = 0.84), attention problems (p = 0.38), aggressive behavior (p = 0.29), rule-breaking behavior (p = 0.11), conduct problems (p = 0.22) and internalizing behavior (p = 0.25) | Maternal education level, occupation of the head of the household, number of children and number of people living together in the house, age and sex, whether parents were living together | Good |
| Rahbar, et al. (2015) [34] | Case-control | Jamaica | n = 200, total population n = 100 with autism n = 100 age and sex-matched controls | 2-8 years | No | Blood | Presence/absence of Autism Spectrum Disorder (ASD^[[25]](#footnote-25)^) | Adjusted concentrations: mean (SD) ASD cases: 2.55 (2.02) µg/dL Controls: 2.72 (2.02) µg/dL | No significant association between blood lead levels and presence of ASD in children | Age, sex place of birth, maternal age, maternal education, paternal age, paternal education, number of children and adults in household, assets owned by the family | Good |
| Rahman, et al. (2002) [35] | Cross-sectional | Pakistan | n = 138 | 6-10 years | No | Blood, teeth | Raven’s Standard Progressive Matrices, school performance assessed by the percentage mark in a school examination, teachers rated the children's behavior | Mean (SD) Blood lead: 16.08 (6.29) µg/dL Tooth lead: 5.68 (4.10) µg/g | Blood lead: negative correlation with IQ (β = -0.430, p = 0.021) but not behavior (univariate regression analysis) or school score Tooth lead: negative correlation with behavior (β = -0.128; p = 0.01) and school score (β = -0.890; p = 0.011) but not with IQ (univariate regression analysis) | Age, height, weight, head circumference, hemoglobin | Good |
| Rasoul, et al. (2012) [36] | Cross-sectional | Egypt | n = 190 | Grades 1, 3, and 5 | No | Blood, environment | Arabic version of the Wechsler Scale | Mean (SD) Blood lead: 6.72 (4.3) µg/dL | Children with blood lead levels > 10 µg/dL have a significantly lower IQ score (88.8 ± 13.49 µg/dL) than children with blood lead < 10 µg/dL (94.72 ± 5.99); p < 0.001 | None found | Good |
| Ruiz-Castell, et al. (2012) [37] | Cohort | Bolivia | n = 246 | 10.5-12.5 months | Yes | Blood | BSID-II | Median (Interquartile Range) 1.76 (2.48-1.34) µg/dL | Cohort had lower measured levels of tested elements than anticipated (all under control limit); was unable to answer primary research questions. Unexpectedly, exposure to low levels of blood lead was positively associated with BSID performance (p <0.05). | Gender, cesarean birth, pregnancy term, maternal age, maternal educational level, parity, marital status, number of adults I the household, hospital of birth, smoking/alcohol in the home, maternal blood lead level | Good |
| Schnaas, et al. (2000) [38] | Cohort | Mexico | n = 112 | 6-month intervals between 36 and 60 months of age | No | Blood | the GCI of the Mc-Carthy Scales of Children's Abilities | Mean 6-18 months: 10.1 µg/dL 24-36 months: 9.7 µg/dL 42-54 months: 8.4 µg/dL | Increasing blood lead at 24-36 months is negatively associated with GCI scores at 48 (p = 0.021) months but not 54 months Increasing blood lead at 42 - 54 months was associated with decreased GCI scores at 54 (p = 0.040) but not 60 months | Sex, apgar score at 5 min, birth weight, birth order, maternal education, family socioeconomic status | Good |
| Schnaas, et al. (2006) [39] | Cohort | Mexico | n = 150 | 5 years | Yes | Blood | Wechsler Intelligence Scale for Children, Revised | Mean (range) Pregnancy: 8.0 (1-33) µg/dL 1-5 years: 9.8 (2.8-36.4) µg/dL 6-10 years: 6.2 (2.2- 18.6) µg/dL | Negative association between blood lead levels at third trimester of pregnancy and FSIQ (β = -3.90, 95% CI: -6.45- -1.36, p = 0.0029) | Maternal IQ, socioeconomic status, degree of stimulation and quality of caretaker-child interaction | Good |
| Solon, et al. (2008) [40] | Cross-sectional | Philippines | n = 877 | 6 months-5 years | No | Blood | BSID-II (6-35 months) WPPSI-III (36-59 months) | Mean blood lead levels: 7.1 µg/dL | A 1 µg/dL increase in blood lead levels was associated with a 3.22 point decline in the MDI in younger children (95% CI: -5.02, -1.6; p < 0.01) and a 2.57 point decline in VIQ (95% CI: -4.58, -0.35; p = 0.02)  Blood lead levels not correlated with PDI or PIQ | Roof material, water source, sex, history of breastfeeding, hemoglobin, age, HOME^[[26]](#footnote-26)^ score, maternal education, maternal smoking, history of prematurity, years of school, region of residence | Good |
| Téllez-Rojo, et al. (2006) [41] | Cross-sectional | Mexico | n = 294 | 12 and 24 months | No | Blood | BSID-II | Mean (SD) 12 months: 4.27 (2.14) µg/dL 24 months: 4.28 (2.25) µg/dL | Blood lead levels at 12 months not associated with MDI or PDI. At 24 months, negatively associated with MDI (β = -4.70, 95% CI: -2.44, -6.97; p < 0.01) and PDI (β = -5.44, 95%CI: -7.35, -3.54, p < 0.01) | Sex, birth weight, maternal IQ | Good |
| Vega-Dienstmaier, et al. (2006) [42] | Cross-sectional | Peru | n = 134 | 6-8.5 years old | No | Blood | Graphic Test of Reasoning, Kohns Block Design Test | Mean (SD) 10.33 (7.36) µg/dL | Children with blood lead > 10 µg/dL presented a greater prevalence of low scores in the Graphic Test of Reasoning (18.9% vs 8.1%, p = 0.059) and in the Kohns Block Design test (39.6% vs 18.6%, p = 0.01) compared to lead < 10 µg/dL | None found | Fair |
| Vigeh, et al. (2014) [43] | Cohort | Islamic Republic of Iran | n = 174 | 3 years | Yes | Maternal and fetal blood | Harold Ireton Early Child Development Inventory | Mean (SD) First trimester: 4.15 (2.43) µg/dL Second trimester: 3.44 (1.28) µg/dL Third trimester: 3.78 (1.40) µg/dL Cord: 2.86 (1.09) µg/dL | Maternal blood lead levels were significantly higher in children with developmental scores <20% than in those with normal scores (6.3± 1.9 vs 4.0± 2.4mg/dL, respectively, P=.01); also lead levels were inversely associated with the development scores (r = –0.155, P = .041). | Hematocrit, maternal education level, body mass index, family income level, completed gestational age, birth order | Good |
| Wang, et al. (1989) [44] | Cohort | China | n = 157 | 6-14 years | No | Blood | Wechsler Intelligence Scale for Children, Revised | Mean (SD) School A, near a battery plant: 22.45 (9.19) µg/dL School B, farther away: 18.36 (10.55) µg/dL Total: 21.10 (10.110 µg/dL | Lead has a negative association with VIQ (F = 18.09; r = -0.5126; p < 0.01,), PIQ (F = 12.99; r = -0.4273; p < 0.01, and FSIQ: F= 17.10; r = -0.4918; p < 0.01) | Age, sex, father and mother’s age at child’s birth, order of pregnancies, birth order, birth weight, perinatal, postnatal, and pregnancy factors, maternal and paternal education and occupational status, per capita income, per capita living area, number of siblings, cousin marriage | Fair |
| Wang, et al. (2009) [45] | Cross-sectional | China | n = 317 | 6-12 years | No | Blood, urine, hair, nails | Revised Raven’s Standard Progressive Matrices, Abbreviated Symptom Questionnaire of Conner's Instruments | Mean (SD) Blood: 7.12 (0.156) µg/dL Urine: 11.7 (1.75) µg/g Hair: 12.5 (2.82) µg/g Nails: 25.3 (2.79) µg/g | Negative correlation between hair lead levels (r = 0.151, p< 0.01) and urinary lead levels (r = 0.132; p < 0.05) and IQ No significant relationship between lead and behavior | Gender, age, village of residence | Good |
| Wasserman, et al. (1998) [46] | Cohort | Yugoslavia | n = 239, total population n = 148 living in Mitrovica, a town heavily exposed to metals n = 145 living in Pristina, an unexposed site | 3 years | Yes | Blood | Child Behavior Checklist | Mean (SD) Mid-pregnancy: 12.6(8.8) µg/dL Umbilical cord: 14.0 (10.2) µg/dL 36 months: 25.8 (19.1) | Cord blood levels were positively associated with anxious-depressed (β = 1.16; p = 0.046) and withdrawn (β = 1.52, p = 0.046) behavior and sleep problems (β = 1.00, p = 0.011).   Blood lead levels at 6 months was positively associated with aggressive (β = 2.11; p = 0.036) and destructive behavior (β = 1.21; p = 0.30)  Blood lead levels at 24 months was positively associated with withdrawn (β = 2.46; p = 0.004) and destructive behavior (β = 1.21; p = 0.23)  Blood lead levels at 30 months was positively associated with withdrawn (β = 2.60; p = 0.013) and destructive behavior (β = 1.37; p = 0.032)  Blood lead levels at 36 months was positively associated with anxious-depressed (β = 1.45; p = 0.044) and withdrawn (β = 3.0; p = 0.001) behavior, sleep problems (β = 1.09; p = 0.024), somatic problems (β = 1.71; p = 0.016), and destructive behavior (β = 2.07; p = 0.001) | Town, sex, ethnicity, maternal education, HOME score, home type | Fair |
| Wasserman, et al. (2003) [47] | Cross-sectional | Yugoslavia | n = 209, total population n = 111 living in Mitrovica, a town heavily exposed to metals n = 98 living in Pristina, an unexposed site | 10-12 years | No | Blood, bone | WISC-III | Mean (SD):  High level village Blood lead: 6.1 (1.9) µg/dL Tibial lead: 1.3 (6.6) µg/g | Blood lead levels negatively associated with VIQ (p < 0.01); PIQ (p < 0.01), and FSIQ (p < 0.05) Tibial lead negatively associated with VIQ (p < 0.01), PIQ (p < 0.01), and FSIQ (P < 0.01) | Age, gender, number of siblings, language spoken in the home, HOME score, maternal Raven score, maternal age, maternal years of schooling | Fair |
| Wolf, et al. (1994) [48] | Cohort | Costa Rica | n = 163 | 12-23 months: BSID, infant behavior record, 5 years: all of the other tests | No | Blood | BSID Infant Behavior Record Woodcock-Johnson Psycho-Educational Battery Bruiniks- Osteretsky Test of Motor Proficiency Beery Developmental Test of Visual motor Integration Goodenough Draw-a-Man WPPSI | Mean (SD) 11 (3.80) µg/dL | Significant positive association with  Gross motor composite subtest from the Bruininks-Oseretsky Test of Motor Proficiency (p = 0.04), picture vocabulary subtest (of the Woodcock-Johnson Psycho-Educational battery) (p = 0.05) No association with  MDI, PDI, Infant Behavior Record, Spatial Relations, Visual Auditory, Quantitative Concepts, Preschool Cluster, Visual Matching, and Perceptual Speed (of the Woodcock-Johnson Psycho-Educational battery), Fine Motor Composite, Battery Composite (of the Bruininks-Osteretsky Test of Motor Proficiency), Beery Developmental test of Visual motor integration, Goodenough Draw-a-Man: (p = 0.17), or any of the subtests of the WPPSI | Family background measures, maternal and paternal IQ, maternal education, maternal weight, maternal height, presence of father or grandparents in the home, HOME score, birth order, gestational age, sex, birth weight, type of delivery, weight, height, intake of cow’s milk, age at weaning from the breast, iron status | Good |

Table D: Summary of the studies involving manganese included in this systematic review

| Author | Study design | Country | Population | Age at outcome measurement | Prenatal measurement | Method of measuring metal exposure | Neurological assessment | Concentration | Results | Covariables adjusted for within the analyses | Quality Assessments |
| --- | --- | --- | --- | --- | --- | --- | --- | --- | --- | --- | --- |
| Βetancourt, et al. (2015) [49] | Cross-sectional | Ecuador | n = 93 | Not specified | No | Hair | Raven’s Colored Progressive Matrices (RCPM^[[27]](#footnote-27)^) | Mean (Standard deviation (SD^[[28]](#footnote-28)^)) Gramadal-Las Vegas: 4.01 (7.5 µg/g) Puyango Viejo: 1 µg/g (SD unspecified) Portovelo-Zaruma (2 µg/g) | Positive correlation between RCPM scores and hair manganese levels (p = 0.03, R^2^ = 0.05) Children > 2 µg/g hair manganese: worst performance (p = 0.004) | Region, gender, age | Good |
| Carvalho, et al. (2014) [50] | Cross-sectional | Brazil | n = 70 | 7-12 years | No | Hair | Vocabulary, Block design, and Digit Span subtests from Wechsler Intelligence Scale for Children (WISC^[[29]](#footnote-29)^)- III  3 attention tasks from the Visual Attention Test  Corsi Block-Tapping Task  Wisconsin Card Sorting Test | Mean (SD):  14.6 (11.8) | Inverse associations between log hair manganese levels and scores on estimated IQ (β = -9.67, 95% confidence interval (CI^[[30]](#footnote-30)^): -16.97, -2.37; p < 0.05), Block Design (β = -2.50; 95% CI: -3.91, -1.10; p < 0.01) and Digit Dpan total (β = -2.59; 95% CI: -4.13, -1.05; p < 0.01) standardized scores and the number of correct answers in forward (β = -1.32; 95% CI: -2.23, -0.40; p < 0.01) and backward (β.= -1.09; 95% CI: -2.02, -0.16; p < 0.05) Digit Span methods  No correlation between hair manganese levels and vocabulary, Corsi Blocks forward, Corsi Blocks backward, reaction time, trials to complete first category, and perseverative errors in the Wisconsin Card Sorting Test | Age, maternal education, family income, height, weight, body mass index, birth weight, place of residence during pregnancy | Good |
| Carvalho, et al. (2018) [51] | Cross-sectional | Brazil | n = 70 | 7-12 years | No | Manganese: Hair Lead: blood | 3 subtests from the Developmental Neuropsychological Assessment Battery Second Edition: Inhibition, Word Generation, and List Memory  Grooved Peg Board Test   Conners Abbreviated Rating scale | Median (range) Hair manganese: 11.5 (0.5-55.7) µg/g Blood lead:  0.5 (0.5-6.1) µg/dL | Log10 hair manganese levels negatively associated with the free recall after interference (β = -1.8, 95% CI: -3.4, -0.2; p = 0.03) and Delayed Effect (β = -2.0; 95% CI: -3.7, -0.2, p = 0.03) from the list memory tests, and the total score from the Conners Abbreviated Scale ( β = -6.6, 95% CI: -12.0, -1.3, p = 0.02)  No significant association with the other tests | Age, sex, school maternal IQ, socioeconomic status | Good |
| Claus Henn, et al. (2010) [52] | Cross-sectional | Mexico | n = 486 | 6, 12, 18, 24, 30, 36 months | No | Blood | Bayley Scales of Infant Development-II | Mean (SD) Blood manganese 12 months: 24.3 (4.5) µg/L 24 months: 21.1 (6.2) µg/L  Blood lead Cord blood: 4.7 (3.1) µg/dL 12 months: 5.1 (2.6) µg/dL 24 months: 4.9 (2.5) µg/dL | An inverted U-shaped association between 12-month blood manganese levels and the Mental Developmental Index (compared with the middle 3 manganese quintiles) for the lowest manganese quintile (< 20.2 µg/L) (β = -3.3, 95% CI: -6.0, -0.7) and for the highest manganese quintile (> 28.0 µg/L) (β = -2.8, 95% CI: -5.5, -0.2), (p = 0.04)  Manganese not significantly associated with the Mental Developmental Index at any other time point | Sex hemoglobin, ferritin, umbilical cord blood lead, birth weight, birth length, head circumference at birth, gestational age, maternal age at delivery, maternal and paternal education, maternal marital status, maternal blood lead at 1 month post-partum, duration of breastfeeding in infant’s first year, child nutrition, maternal IQ | Good |
| Menezes- Filho, et al, (2014) [53] | Cross-sectional | Brazil | n = 70 | 7-12 years | No | Manganese: hair Lead: blood | Child Behavior Checklist, Portuguese version | Mean (SD): Hair manganese Boys: 15.3 (9.9) µg/g Girls: 13.9 (13.2) µg/g  Blood lead Boys: 1.2 (1.3) µg/dL Girls: 1.1 (0.9) µg/dL | Positive correlation between Log hair manganese and total Child Behavior Checklist score (β = 13.99, 95% CI: 3.06- 24.94; p = 0.013), indicative of poor behavior. There is also increased externalizing behavior (β = 7.57, 95% CI: 2.14, 13.00; p = 0.007), and attention problems (β = 2.97; 95% CI: 0.78- 5.17; p = 0.009) | Age, sex, maternal IQ | Good |
| Menezes-Filho, et al. (2011) [54] | Cross-sectional | Brazil | n = 83 | 6-12 years | No | Manganese: hair, blood Lead: blood | WISC-III | Mean (SD) Mother's hair manganese: 3.5 (12.76) µg/g  Child hair manganese: 5.83 (11.5) µg/g Child blood manganese: 8.2 (3.6) µg/g Child blood lead: 1.43 (1.90) µg/dL | Children's log hair manganese negatively correlated with the Verbal Intelligence Quotient (β = -6.92; 95% CI: -11.81, -0.63); and the Full Scale Intelligence Quotient (β = -5.78; 95% CI: -10.71, -0.21)   Blood manganese not correlated with IQ | Age, sex, height, weight, body mass index, ethnicity, marital status, maternal education, maternal age at birth, maternal Raven score, family income, HOME^[[31]](#footnote-31)^ score, number of children in the home, serum iron, blood lead, maternal hair manganese | Good |
| Nascimento, et al (2016) [55] | Cross-sectional | Brazil | n = 63, total population living in Rio Grande do Sul, Southern Brazil n = 43, living in a rural area n = 20, living in an urban area | 6- 12 years | No | Water, blood, urine | Brazilian Child Brief Neuropsychological Assessment Battery, RCPM | Mean (SD) Rural area:  Blood: 32.8 (3.47) µg/L Hair: 2.17 (2.63) µg/g Water: 18.5 (35.41) µg/L  Urban area Blood:: 19.25 (6.36) µg/L Hair:: 0.47 (.33) µg/g Water: 1.3 (.66) µg/L | Blood manganese negatively associated with visual attention (β = 0.649, p < 0.001), visual perception (β = -0.472, p = 0.014) and phonological awareness (β = -0.360; p = 0.029), but not with visuospatial working memory (span), word/nonword reading, word/nonword written, written language (literacy) total score, language (total score), and the go/no go task   Hair manganese negatively associated with the visuospatial working memory (β = -0.377; p = 0.0399) but not with the other tests   Water manganese negatively associated with word/nonword reading (β = -0.307, p = 0.019), word/nonword written (β = -0.253; p = 0.045), written language (literacy) (total score) (β = -0.361, p = 0.007), language (β = -0.390; p = 0.002), and go/no go task (executive functions- inhibition) (β = -0.547; p = 0.001) | IQ, age, sex, maternal and paternal education | Good |
| Rahbar, et al. (2014) [56] | Cross-sectional | Jamaica | n = 218, total population n = 109 Autism Spectrum Disorder (ASD^[[32]](#footnote-32)^) cases n = 109 matched controls | 2-8 years | No | Blood | Presence/absence of ASD | Mean (SD) ASD: 10.9 (2.8) µg/L Control: 10.6 (2.8) µg/L | No significant correlation between blood manganese and ASD | Diet, parental education, paternal age, place of child’s birth | Good |
| Rahman, et al. (2017) [57] | Case control | Bangladesh | n = 1265 | 10 years | Yes | Drinking water | WISC-IV, Strength and Difficulties Questionnaire | Median, (5th, 95th percentile):  Prenatal Manganese: 204 (23, 2494) µg/L Arsenic: 33 (0.1, 412) µg/L  5 years Manganese: 228 (8.2, 2605) µg/L Arsenic: 3.6 (0.1, 266) µg/L  10 years:  Manganese: 339 (4.0, 3203) µg/L Arsenic: 2.2 (0.1, 311) µg/L | Water manganese, multivariable adjusted model: not significantly associated with WISC; hyperactivity, peer problems, emotional problems, or prosocial behavior  Water manganese positively associated with conduct problems at all time points (Prenatal water manganese: (β = 1.20; 95% CI: 1.04 - 1.39; p = 0.013); 5 years: (β = 1.2, 95% CI = 1.03 - 1.39; p = 0.016); 10 years: (β = 1.17, 95% CI = 1.04- 1.31; p = 0.007))  Prenatal water manganese associated with decreased risk of emotional problems (Odds ratio (OR^[[33]](#footnote-33)^) = 0.39, 95% CI: 0.19, 0.82) in boys and low prosocial scores (OR: 1.48; 95% CI: 1.06, 1.88) in girls | Age, gender, height, education, type of school attended, hemoglobin, maternal IQ, socioeconomic status, number of siblings, HOME score, testers of cognitive abilities, urinary arsenic concentration | Good |
| Torres-Agustin, et al. (2013) [58] | Cohort | Mexico | n = 174, total population living in Hildago State n = 79, living in Molango, a mining area n = 95, living in Agua Blanca, a nonmining area | 7-11 years |  | Manganese: Hair, blood Lead: blood | Children’s Auditory Verbal Learning Test | Median (range) Mining district Hair manganese: 12.6 (4.2-48) µg/g Blood manganese: 9.5 (5.5-18.0) µg/L Blood lead: 3.3 (0.5- 13.5)  Nonmining district:  Hair manganese: 0.6 (0.06-3.6) µg/g Blood manganese: 8.0 (5.0-14.0) ug.L Blood lead: 8.0 (1.8-22.5) | Hair manganese negatively associated with level of learning (β = -0.47; 95% CI: -0.84, -0.09; p = 0.016) and immediate recall (β = -0.41; 95% CI: -0.78, -0.03; p = 0.033) but not with the immediate memory span, delayed recall, or recognition accuracy, subtests of the Children’s Auditory Learning Test  Blood manganese is not significantly associated with any of the subtests | Sex, age, blood lead, hemoglobin, maternal education | Good |
| Yu, et al. (2014) [59] | Cross-sectional | China | n = 933 | 3 days | No | Umbilical cord serum | Neonatal behavioral neurological assessments (NBNA^[[34]](#footnote-34)^) | Median: 4.0 µg/L | Higher level of manganese (≥ 9.1 µg/L) associated with lower NBNA scores (β = -1.2, 95% CI: -1.4, -1.0; p < 0.001) and a higher risk of low NBNA score (OR = 13.0, 95% CI = 6.3, 26.6, p < 0.01) Cord serum manganese ≥ 50 µg/L: adverse effects on behavior, active tone, general reactions of clusters ( p < 0.001) | Maternal age, family income, maternal and paternal occupation, diet | Good |

Table E: Summary of the studies involving mercury included in this systematic review

| Author | Study design | Country | Population | Age at outcome measurement | Prenatal measurement | Method of measuring metal exposure | Neurological assessment | Concentration | Results | Covariables adjusted for within the analyses | Quality assessment |
| --- | --- | --- | --- | --- | --- | --- | --- | --- | --- | --- | --- |
| Chevrier, et al. (2009) [60] | Cross-sectional | Brazil | n = 253, total sample n = 65 in Sai Cinza, on the Tapajos River Tributary n = 94 in Brasilia Legal, on the Tapajos river tributary n = 94 Santana do Ituqui, on the main amazon river | 7-12 years | No | Hair | Stanford-Binet Copying test | Mean (range) Sai Cinza, closest village to the gold-mining areas: 19.4 µg/g (9.6-63.8) Santana do Ituqui: far from pollution sources: 5.5 µg/g (0.5-12.4) Total sample: 9.8 (0.5-63.8) | Child hair mercury, log 10 scale: negatively associated with relaxed copying score (β = -0.96; p = 0.009) and block score (β = -2.27; p < 0.001)  Mother hair mercury, log 10 scale: negatively associated with relaxed copying score (β = -0.80; p = 0.05) and block score (β = -2.24, p < 0.001) | Age, sex, village, marital status, maternal Raven score, maternal alcohol consumption during pregnancy | Good |
| Counter, et al. (2006) [61] | Cross-sectional | Ecuador | n = 73, total population n = 53 living in the gold mining settement of Nambija n = 20, living in the gold minng town of Portovelo | 5-11 years | No | Blood, urine, and hair | Raven’s Colored Progressive Matrices | Mean (standard deviation (SD^[[35]](#footnote-35)^)):  Blood: 5.1 (2.4) µg/L Urine: 13.3 (25.9) µg/L Hair: 8.5 (22.8) µg/L | Children with hair mercury > 2 µg/g and blood mercury > 5 µg/L had significantly lower scores on the Raven’s Colored Progressive Matrices test than did children with nontoxic mercury exposure levels (t = -2.16, p < 0.034) | None found | Good |
| Dorea, et al. (2012) [62] | Cross-sectional | Brazil | n = 281, total population n = 33, living in Itapua, former subsistence fisherman families n = 166, living in Bom Futuro, families of Casserite Settlers n = 82, living in Porto Velho, a gold mining town | 1-6 months | No | Hair | Gesell Developmental Schedules (GDS^[[36]](#footnote-36)^) | Mean (SD) Itapua: Hair: 4.96 (1.8) µg/g  Bom Futuro Hair 1.85 (0.9) µg/g  Porto Velho Hair: 3.84 (5.5) | Inverse correlation between mercury and GDS scores only in Porto Velho (Spearman r = -0.2300, p = 0.0376); but not in Bom Futuro (Spearman r = 0.1336; p = 0.0862) or Itapua (spearman r = 0.1666; p= 0.0862) | Frequency of family fish consumption, breastfeeding, presence of thiomersal-containing vaccines | Good |
| Dorea, et al. (2014) [63] | Cross-sectional | Brazil | n = 299, total population n = 91, living in Itapua n = 218, living in Bom Futuro | 12-24 months | No | Hair | GDS | Median (range) Itapua Hair 3.5 (1.0-8.7) µg/g   Bom Futuro: Hair: 2.2 (0.5-8.6) µg/g | No significant association between hair mercury levels and GDS score | Age at visit, age at walking, age at talking, hemoglobin, weight, height, head circumference, number of children, presence of thiomersal containing vaccines, umber of fish meals per week, breastfeeding duration, family income, maternal education level | Good |
| Fonseca, et al. (2008) [64] | Cross-sectional | Brazil | n = 70, total sample n = 32, Luna, Espirito Santo: low mercury n = 38, Puruzinho Lake Community in the Rio Madeira Basin: high mercury | 6.5-15.5 years | No | Hair | Wechsler Intelligence Scale for Children (WISC^[[37]](#footnote-37)^)-III, human figure drawings | Mean (SD) Luna: 0.28 (0.22) Puruzinho: 18.4 (12.76) | No significant correlation between mercury level and WISC score or human figure drawings | Age, sex, school level, height, weight, body mass index | Good |
| Gao, et al. (2007) [65] | Cross-sectional | China | n = 408 in Zhoushan city, an important costal fishery n = 203 males n = 205 females | 3 days | No | Maternal hair samples, cord blood | Neonatal behavioral neurological assessments | Mean (Interquartile range (IQR^[[38]](#footnote-38)^)): Maternal hair: 1246.56 µg/Kg (927.34-1684.67) Cord blood: 5.58 µg/L | Negative association between mercury level and behavior, but only in males (Odds ratio (OR^[[39]](#footnote-39)^) = 1.235, 95% confidence interval (CI^[[40]](#footnote-40)^) of OR = 1.078 - 1.414, P < 0.001) No significant association was found for females | Sex, apgar score at 5 min, birth order, birth weight, body height, head circumference, Fish intake, type of fish consumed during pregnancy, whether dental treatment was received during pregnancy, maternal education, maternal occupation, presence of a cold during pregnancy, anemia during pregnancy, premature rupture of membranes, monthly household income per capita, paternal smoking, paternal alcohol consumption, length of stay in region | Good |
| Grandjean, et al. (1999) [66] | Cross-sectional | Brazil | n = 351 living along the Tapajos river, Brazil n = 105: Santana do Ituqui n = 91: Brasilia Legal n = 87: Sao Luis do Tapajos n = 71: Sai-Cinza | 7-12 years | No | Maternal child hair samples | Finger tapping task Santa Ana Form Board Digit Spans subtest from the WISC-III  Stanford Binet Intelligence test | Mean: Children: 11.6 µg/g Mothers: 11.0 µg/g | Mercury was only negatively correlated with the preferred hand (β = -4.57; p = 0.002), other hand (β = -4.12, p = 0.001) and both hands (β = -5.58; p = 0.001) in the Santa Ana test and the Copying (β = -3.40; p = 0.003) and Recall (β = -1.23, p = 0.02) subtests of the Stanford-Binet Intelligence test | Age, sex, village | Good |
| Gustin, et al. (2017) [67] | Cross-sectional | Bangladesh | n = 1434 | 10 years | No | Hair | WISC-IV, Parent-report Strengths and Difficulties Questionnaire | Median (range): 0.674 µg/g (0.07-17.561) | Highest tertile of hair mercury levels (median: 1142 µg/kg): lower prevalence of hyperactivity (OR: 0.61, 95% CI: 0.45- 0.83) and peer relationship problems (OR: 0.58, 95% CI: 0.44-0.76) compared to the lowest tertile (median: 395 µg/kg) No significant association with FSIQ | Weight, height, years of formal schooling, paternal education, maternal age, maternal weight, maternal height, children’s anthropometry at birth, maternal IQ, socioeconomic status, HOME^[[41]](#footnote-41)^ score, selenium and arsenic levels | Good |
| Hu, et al. (2016) [68] | Cohort | China | N = 410 | 1 year | Yes | Maternal and umbilical blood samples | GDS | Mean:  Maternal: 0.72 µg/L Umbilical: 1.2 µg/L | A log-unit increase in umbilical blood mercury levels was associated with a 4.22 point (95% CI: 0.77 to 7.67) increase in the adaptive domain and a 4.06 point (95% CI: 0.51 to 7.62) increase in the social domain of the GDS | Gestational age, parity, sex, birth weight, head circumference, maternal pre-pregnancy body mass index, pregnancy weight gain, frequency of total fish consumption, maternal age, passive smoking during pregnancy, household monthly income, maternal blood lead, maternal blood manganese | Fair |
| Marques, et al (2016) [69] | Cohort | Brazil | n = 1668 | 6 and 24 months | No | Maternal hair | Bayley Scales of Infant Development (BSID^[[42]](#footnote-42)^)- II | Mean (SD) Low exposure 4.87 (2.52) µg/g  Medium exposure 9.85 (11.12) µg/g | Mercury is negatively associated with the Mental Developmental Index (MDI^[[43]](#footnote-43)^) (F value: 3.28; p = 0.0381) but not with the Psychomotor Developmental Index (PDI^[[44]](#footnote-44)^) | Sex, breastfeeding duration, maternal education | Good |
| Marques, et al. (2007) [70] | Cohort | Brazil | n = 82 | 6 months | No | Hair and blood | GDS | Median (range):  umbilical cord blood: 7.44 (0.12-43.74) ng/g Placenta: 8.10 (0.37-56.28) ng/g Blood: 0.55 (0.01-9.97) µg/L Hair: 5.4 (0.39-62.43) µg/g Fetal hair: 1.59 (19.65-0.05) µg/g 6 months: 1.81 (0.02-32.95) µg/g | No significant association between mercury level and GDS score | Maternal education, income, type of home, type of water supply, amount of fish meals per week, fish-eating sources | Good |
| Marques, et al. (2009) [71] | Cohort | Brazil | n = 82 | 6, 36, and 60 months | No | Hair | GDS | Not specified | Negative association between postnatal mercury and GDS scores at 5 months (r = -0.333, p = 0.002) and 60 months (r = -0.803; p = 0.010) | Day of first thiomersal-containing vaccine, gestational age, infant body mass percent increase at 6 months, length of breastfeeding | Good |
| Marques, et al. (2010) [72] | Cohort | Brazil | n = 82 | 6 months | No | Maternal and fetal hair at birth | GDS | Mean (range): Unexposed to vaccines:  Fetal hair 2.1 (0.1-9.3) µg/g Maternal hair 10.5 (0.8-62.4) µg/g   Exposed: Fetal hair 2.5 (0.1-19.6) µg/g Maternal hair: 6.4 (0.4-28.8) µg/g | Child mercury levels at birth negatively associated with GDS for most components: motor (β = -0.357l; p = 0.001), language ( β = -0.259; p = 0.02), adaptive: (β = -0.376; p = 0.000) and general (β = -0.344; p = 0.002) scores  Child mercury levels at 6 months negatively associated with language (β = -0.288; p = 0.009) ,adaptive (β = -0.292; p = 0.007), and general (β = -0.252; p = 0.020)  Maternal mercury at birth or 6 months not significantly associated with any subtest | Whether mothers received thiomersal-containing vaccines during pregnancy | Good |
| Marques, et al. (2012) [73] | Cross-sectional | Brazil | n = 688 | 1-59 months | No | Hair | GDS | Mean (SD) Hair: 2.28 (1.15) µg/g | Significant correlation between family fish consumption and mercury levels (Spearman r = 0.1756; p < 0.0001), and family fish consumption and GDS score (Spearman r = 0.080, p = 0.035) | Sex, age, birth weight, weight, height, head circumference, maternal schooling, breastfeeding duration, presence of thiomersal-containing vaccines | Good |
| Marques, et al. (2015) [74] | Cohort | Brazil | n = 294 | 6 months and 24 months | No | Hair | BSID-II | Mean (SD) Girls: birth: 0.81 (0.33) µg/g 6 months: 1.02 (0.3) µg/g 24 months: 5.48 (4.01) µg/g   Boys:  birth: 0.81 (0.31) µg/g 6 months: 1.01 (0.28) µg/g 24 months: 1.87 (0.76) µg/g | Neonatal mercury negatively associated with MDI (β = -0.222, 95% CI: -0.44 - 0.01; p = 0.045) at 24 months in boys  Mercury not significantly associated with MDI or PDI at any other time point | Sex, birth weight, family income, maternal education, breast-feeding length | Good |
| Marques, et al. (2016) [75] | Cohort | Brazil | n = 365, total population n.= 132 with home delivery n = 233 with hospital delivery | 6 months: BSID-II 60 months: Stanford-Binet | No | Hair | BSID-II Stanford-Binet Intelligence test | Mean (SD) Home delivery: Mother: 23.9 (20.1) µg/g Child: 7.13 (3.5) µg/g   Hospital delivery Mother 12.2 (12.7) µg/g Child 4.59 (2.8) µg/g | Hospital delivery: higher mercury levels (p < 0.0001) than home. No significant association between place of delivery and any of the subtests except for an increased score in the fluid reasoning subtest of the Stanford-Binet test in hospital deliveries (p = 0.001) | Birth weight, birth length, weight and height at time of measurement, age at walking, age at talking, Maternal age, number of children, maternal education, length of breastfeeding | Good |
| Marques, et al. (2016) [76] | Cross-sectional | Brazil | n = 690, population n = 258, breastfed for 6 months n = 288, breastfed for 6- 12 months n = 144, breastfed for up to 24 months | 6 and 24 months | No | Hair | BSID-II | Mean (SD) Short breastfeeding duration:  Mother 12.6 (17.0) µg/g Child 2.7 (2.6) µg/g   Medium breastfeeding duration  Mother: not measured Child: not measured  Long breastfeeding duration Mother: 9.8 (10.7) Child: 4.1 (2.1) | Maternal mercury: positive correlation with MDI (DF = 4; F value: 7.58; Pr > F: < 0.0001) and PDI (DF: 4; F value: 3.29; Pr> F: 0.0124) | Duration of breastfeeding, birth weight, number of children, maternal age, maternal education, age at walking and talking | Good |
| Rothenberg, et al (2021)  [77] | Cohort | China | n = 190 | 12 months and 36 months | Yes | Hair | BSID - II | Median (range): 0.42 (0.12-1.7) µg/dL | Doubling the maternal hair mercury concentration resulted in a 1.3 point decrease in the MDI score (95% CI: -2.6, -0.14) and a 1.2 point decrease in the PDI score (95% CI -2.6, 0.14) | Maternal age, maternal fish consumption, maternal rice consumption, maternal serum zinc, maternal blood lead, maternal energy intake, pre-pregnancy body mass index, maternal education, child sex, child’s age at testing, birth weight for gestational age | Good |
| Tavares, et al. (2005) [78] | Cross-sectional | Brazil | n = 209, total sample living alongside Rio Cuiaba n = 75: High dependency for fish n = 134: Low dependency | 3-7 years | No | Hair | Lefevre Evolutional Neurological Test | Mean (SD) High fish consumption: 5.37 (3.35) µg/g Low fish consumption: 2.08 (1.37) µg/g | No significant association between mercury level and any of the subtests | None found | Good |

Table F: Summary of the studies involving metal mixtures in this systematic review

| Author | Study design | Country | Population | Age at outcome measurement | Prenatal measurement | Metal | Method of measuring metal exposures | Neurological assessment | Concentration | Results | Covariables adjusted for within the analyses | Quality assessment |
| --- | --- | --- | --- | --- | --- | --- | --- | --- | --- | --- | --- | --- |
| Bao, et al. (2009) [79] | Cross-sectional | China | n = 549 | 7-16 years | No | Cadmium, copper, lead, zinc | Water: cadmium, lead, copper, zinc  Hair: cadmium, lead, zinc | Child Behavior Checklist | Excretion water:  Cadmium: 7.09x10^-3^ mg/L Lead: 0.043 mg/L Copper: 0.196 mg/L Zinc: 13.700 mg/L  Hair: mean (range) Cadmium: 0.10 (0.01-6.85) Lead: 4.19 (0.08-58.24) Zinc: 211.52 (32.71-1295.99) | Lead positively associated with anxious/depressed (β: 0.894; standard error (SE^[[45]](#footnote-45)^): 0.111; p < 0.001) and withdrawn (β: 0.410; SE: 0.081; p = 0.000) moods, somatic complaints (β: 0.743; SE: 0.105; p = 0.000) social (β = 0.676; SE: 0.099; p = 0.000), thought (β = 0.856; S: 0.104; p = 0.000) and attention problems (β: 0.606; SE: 0.089; p = 0.000 and delinquent (0.752; SE: 0.095; p = 0.000) and aggressive behavior (β = 1.207; SE: 0.162; p = 0.000)  Cadmium positively associated with withdrawn mood (β: 0.282; SE: 0.088; p= 0.001); social problems (β: 0.274; SE: 0.108; p = 0.012), but not with anxious/depressed moods, somatic complaints, thought and attention problems or delinquent and aggressive behavior.   Zinc negatively associated with anxious/depressed (β = -1.180; SE: 0.212, p = 0.000), withdrawn (β = -0.792; S: 0.154; p = 0.000) moods, somatic complaints (β = -0.709; SE: 0.200; p = 0.000), social (β = -1.053; SE: 0.190; p = 0.000), thought (β = -0.729; SE: 0.198; p = 0.000); and attention problems (β = -0.660; SE: 0.169; p = 0.000), and delinquent (β = -0.641; SE: 0.181; 0.000) and aggressive behavior (β = -1480; SE: 0.309; p = 0.000) | Sex, age, towns, parental education | Good |
| Bora, et al. (2019) [80] | Cross-sectional | Democratic Republic of the Congo | n = 95 | 6- 11 years | No | Selenium, iron, chromium, copper, zinc, manganese, arsenic, lead, cadmium, copper | Blood, urine | Kaufman assessment battery for children, 2nd edition | Mean (standard deviation (SD^[[46]](#footnote-46)^)) Blood:  Selenium: 70.7 (16.1) µg/l Iron: 2573.8 (9354.7) µg/L Chromium: 0.3 (2.5) µg/L Copper: 1328.5 (331.5) µg/L Zinc: 592.5 (256.9) µg/L Manganese: 1.7 (4.7) µg/L Arsenic: 0.4 (2.5) µg/L Lead: 1.1 (5.8) µg/L Cadmium: 0.02 (0.1) µg/L Co: 4.4 (4.1) µg/L  Urine Selenium: 21.7 (22.2) µg/L Iron: 137.8 (557.6) µg/L Chromium: 0.7 (2.3) µg/L Copper: 62.8 (201.9) µg/L Zinc: 495.1 (352.1) µg/L Manganese: 156 (731.9) µg/L Arsenic: 39.5 (42.5) µg/L Lead: 9.2 (10.4) µg/L Cadmium: 0.6 (0.4) µg/L Cobalt: 15 (19.9) µg/L Mercury: 0.6 (0.8) µg/L | Urinary copper> 5 µg/L associated with poor simultaneous processing (memory) (p = 0.0237);   Higher excretion of zinc had negative influence on the Mental Processing Index (p = 0.020 or Non-Verbal Index (p = 0.01)  Urinary zinc is negatively associated with simultaneous processing (p = 0.02) and planning (p = 0.05)   Urinary arsenic is negatively associated with simultaneous processing ( p = 0.03)  Higher excretion of copper associated with better simultaneous (p = 0.04), the Mental Processing Index ( p = 0.05) and Non-Verbal Index (p = 0.04)  Higher excretion of cadmium is associate with better memory (estimate = 6.9; SE = 3.02; p = 0.03) and planning (estimate: 7.02; SE: 0.05; p = 0.03), as well as the Mental Processing Index (estimate: 7.06; SE: 2.2; p< 0.01) and Nonverbal Index (estimate: 7.92; SE: 2.87; p = 0.012) | Sex, age, socioeconomic status | Good |
| Calderón, et al. (2001) [81] | Cross-sectional | Mexico | n = 80, total population n = 41 exposed to high levels of Arsenic and lead n = 39 exposed to low levels of Arsenic and lead | 6- 9 years | No | Arsenic, lead | Arsenic: urine  Lead: blood | Wechsler Intelligence Scale for Children, Revised Mexican Version | Mean (SD)  Exposed group Arsenic: 62.9 (0.03) ug arsenic/g creatinine Lead: 8.9 (0.03) µg/dL   Unexposed group: Arsenic: 40.2 (0.03) ug arsenic/g creatinine Lead 9.7 (0.02) µg/dL | Urinary arsenic negatively correlated with the Verbal Intelligence Quotient (VIQ^[[47]](#footnote-47)^) (r = -0.43; p = 0.008), and the Full Scale Intelligence Quotient (FSIQ^[[48]](#footnote-48)^) (r = -0.33, p = 0.03), and the concepts (r = -0.31; p < 0.05), knowledge (r = -0.41; p < 0.05), sequential (r= -0.31; p < 0.05) subtests only for the children in the exposed population. There is no significant association between urinary arsenic and the performance, or spatial subtests.   Blood lead negatively correlated with sequential subtests in the exposed (r = -0.36, p < 0.05) and unexposed (r = -0.30, p< 0.05) population, but not with the concepts, knowledge, spatial, verbal, or performance subtests, and with the FSIQ. | Age, socioeconomic status, maternal and paternal education, height, transferrin saturation | Good |
| Claus Henn, et al. (2012) [82] | Cohort | Mexico | n = 486 | 6-month intervals from 12 to 36 months | No | Lead and manganese | Blood | Bayley Scales of Infant Development (BSID^[[49]](#footnote-49)^)-II | Mean (SD) Manganese 12 months: 24.7 (5.9) µg/L 24 months: 21.5 (7.4) µg/L  Lead Umbilical cord: 4.7 (3.1) µg/dL 12 months: 5.1 (2.6) µg/dL 24 months: 5.0 (2.9) µg/dL Maternal whole blood lead: 7.5 (4.6) µg/dL | Highest manganese quintile was negatively associated with the Mental Developmental Index (MDI^[[50]](#footnote-50)^) (β = -1.27, 95% confidence interval (CI^[[51]](#footnote-51)^): -2.18, -0.37) and Psychomotor Developmental Index (PDI^[[52]](#footnote-52)^) (β = -0.92 (95% CI: -1.76; -0.09)  There is a significant manganese- lead interaction for children in the highest quintile of 12-month blood manganese (adjusted β = -1.27; 95% CI: -2.18 to -0.37) "Given these estimates, we expect MDI scores to decline by 0.07 points per 1 µg/dL increase in lead among children with midrange manganese levels compared with a decline of 2.23 points per 1 µg/dL increase in lead among children with high manganese levels) | Sex, hemoglobin, gestational age, maternal IQ, maternal education | Good |
| do Nascimento, et al. (2015) [83] | Cross-sectional | Brazil | n = 69, total population living in the central region of Rio Grande do Sul, Southern Brazil  n = 43 rural children n = 23 urban children | 6-12 years | No | Lead, arsenic, chromium, manganese, iron | Blood, serum, hair, and drinking water samples | Raven’s Colored Progressive Matrices (RCPM^[[53]](#footnote-53)^) | Mean (SD) Rural children Whole blood Lead: 12.0 (5.6) µg/L Arsenic: 16.0 (1.3) µg/L Mercury: 3.5 (3.3) µg/L Chromium: 19.0 (1.9) µg/L Manganese: 16.0 (4.2) µg/L Iron: 487 (39.9) µg/L  Serum Lead: not measured Arsenic: 14.0 (2.6) µg/L Mercury: 2.7 (3.0) µg/L Chromium: 12.0 (2.2) µg/L Manganese: 2.0 (0.8) µg/L Iron: 1840 (600) µg/L  Hair Lead: 1.4 (2.3) µg/g Arsenic: 0.05 (0.03) µg/g Mercury: 0.08 (0.04) µg/g Chromium: 0.36 (0.1) µg/g Manganese: 2.07 (2.6) µg/g Iron: 18.3 (10.2) µg/g  Water Lead: 0.002 (0.003) mg/L Arsenic: 0.0017 (0.002) mg/L Mercury: 0.001 (0.00001) mg/L Chromium: 0.001 (0.001) mg/L Manganese: 0.02 (0.05) mg/L Iron: 0.31 (0.5) mg/L  Urban children Whole blood Lead: 12.0 (5.0) µg/L Arsenic 16.9 (2.5) µg/L Mercury: 1.1 (0.3) µg/L Chromium: 17.0 (3.7) µg/L Manganese:19.0 (4.3) µg/L Iron: 339 (45) µg/L  Serum Lead: not measured Arsenic: 25.0 (4.0) µg/L Mercury: 1.1 (0.3) µg/L Chromium: 15.0 (1.1) µg/L Manganese: 15.0 (1.0) µg/L Iron: 1870 (250) µg/L  Hair Lead: 1.2 (1.4) µg/g Arsenic: 0.03 (0.01) µg/g Mercury: 0.07 (0.03) µg/g Chromium: 0.43 (0.1) µg/g Manganese: 0.45 (0.2) µg/g Iron: 10.5 (3.1) µg/g  Water Lead: 0.001 (0.0003) mg/L Arsenic: 0.0001 (0.00001) mg/L Mercury: 0.001 (0.00001) mg/L Chromium: 0.001 (0.0002) mg/L Manganese: 0.0001 (0.0007) mg/L Iron: 0.072 (0.05) mg/L | Negative association between hair manganese and RCPM score (β = -0.399, p = 0.012) and between water manganese and RCPM scores (r = -0.317, p = 0.014)  Negative association between hair iron and RCPM score (β = -0.361; p = 0.025)  No significant correlation between the other metal measurements and RCPM score | Age, sex, parental education | Good |
| Hernandez-Bonilla, et al. (2016) [84] | Cross-sectional | Mexico | n = 267, total population n = 148 living in Molango, a mining area n = 119, living in Agua Blanca, a nonmining area | 7- 11 years | No | Manganese, lead | Manganese: hair  Lead: blood | Wechsler Intelligence Scale for Children, Revised Mexican Version | Mean (SD)  Exposed group Arsenic: 62.9 (0.03) ug arsenic/g creatinine  Lead: 8.9 (0.03) µg/dL   Unexposed group: Arsenic: 40.2 (0.03) ug arsenic/g creatinine Lead: 9.7 (0.02) µg/dL | Urinary arsenic negatively correlated with VIQ (r = -0.43; p = 0.008), and FSIQ (r = -0.33, p = 0.03), and the concepts (r = -0.31; p < 0.05), knowledge (r = -0.41; p < 0.05), sequential (r= -0.31; p < 0.05) subtests only for the children in the exposed population. There is no significant association between urinary arsenic and the performance, or spatial subtests.   Blood lead negatively correlated with sequential subtests in the exposed (r = -0.36, p < 0.05) and unexposed (r = -0.30, p< 0.05) population, but not with the concepts, knowledge, spatial, verbal, or performance subtests, and with the full-scale IQ score. | Age, sex, hemoglobin, motor dexterity, maternal Raven score | Good |
| Khaled, et al. (2016) [85] | Case control | Egypt | n = 100, total population n = 40 children with Autism Spectrum Disorder (ASD^[[54]](#footnote-54)^) n = 40 controls n = 20 siblings of children with ASD | 3-6 years | No | Lead, mercury | Blood | DSM-IV-TR, ADI-R, CARS test | Mean (SD) ASD:  Mercury: 32.9 (16.4) µg/L Lead: 16.4 (7.05) µg/L  Controls Mercury: 12.08 (4.5) µg/L Lead: 10.8 (3.4) µg/L  Siblings Mercury: 12.08 (4.5) µg/L Lead 10.8 (3.4) µg/L | Mercury and lead levels significantly higher in those with ASD compared to healthy controls and siblings of children with ASD (32.9 ± 16.4 µg/L vs 12.08 ± 4.5 µg/L and 12.08 ± 4.5 µg/dL; p <0.001 and 16.4 ± 7.05 µg/L vs 10.08 ± 3.4 and 10.08 ± 3.4; p < 0.001 µg/L), respectively | Age, sex | Fair |
| Khan, et al. (2011) [86] | Cross-sectional | Bangladesh | n = 201 | 8-11 years | No | Manganese, arsenic | Manganese: water and blood  Arsenic: water, urine, and blood  Lead: Blood Chromium: Urine | Child Behavior Checklist, Teacher's Report form | Mean (SD)  Water: Arsenic: 43.7 (67.0) µg/L Manganese: 889.2 (783.3) µg/L  Urine Arsenic: 81.2 (75.2) µg/dL Chromium: 33.9 (22.9) mg/dL  Blood Arsenic: 5.1 (3.3) µg/L Manganese: 15.1 (3.9) µg/L Lead: 120.3 (36.4) µg/L | Log-transformed water manganese positively and significantly associated with internalizing (estimated β = 0.82; 95% CI: 0.08 to 1.56; p = 0.03), externalizing (estimated β = 3.35, 95% CI = 0.81 to 4.37; p = 0.004); and total scores (estimated β = 3.35, 95% CI = 0.86, 5.83; p = 0.008)  No significant associations between water arsenic and internalizing (estimated β = -0.29; 95% CI. = 0.65, 0.07), externalizing (estimated β = -0.45; 95% CI = -1.62, 0.73; p = 0.46) or total scores (estimated β = -0.78, 95% CI = -2.18, 0.62, p = 0.28) | Sex, body mass index, arm circumference, maternal education | Good |
| Khan, et al. (2012) [87] | Cross-sectional | Bangladesh | n = 840 | 8-11 years | No | Manganese, arsenic | Manganese: water, blood  Arsenic: water, urine, blood | Annual scores from school in Bangla, English, and Math | Mean (SD):  Water arsenic: 119.5 (147.5) µg/L Water manganese: 1387.9 (866.3) µg/L Urinary arsenic: 138.9 (133.0) µg/L | Water manganese above the World Health Organization standard of 400 µg/L associated with a 6.37% score loss in mathematics (95% CI: -12.27, -0.46; p < 0.05), not with Bangla language score (β = -1.01, 95% CI: -6.14, 4.12) or English language score (β = -2.66, 95% CI: -7.16, 1.83)  Water arsenic not associated with Bangla language score (β = -1.71, 95% CI: -4.77, 1.34), English language score (β = -0.73, 95% CI: -4.32, 2.86) or math score (β = 0.56, 95% CI: -2.98, 4.10) | School grade, head circumference, maternal and paternal education | Good |
| Kippler, et al. (2012) [88] | Cohort | Bangladesh | n = 1305 | 5 years | Yes | Cadmium, lead, and arsenic | Urine | Wechsler Preschool and Primary Scale of Intelligence, Strengths and Difficulties Questionnaire | Median (5th-95th percentiles) Maternal urine (GW8) Cadmium: 0.63 (0.18-2.0) µg/L Arsenic: 86 (18-524) µg/L Lead 2.8 (1.1-6.8) µg/L Exposure at 5 years:  Cadmium: 0.22 (0.078- 0.63) µg/L Arsenic: 53 (17-364) µg/L Lead: 3.8 (1.6-11) µg/L | Maternal and concurrent U-Cd inversely associated with VIQ (β = -1.5; 95% CI: -2 to -1.1; p < 0.001; β = -1.2; ; 95% CI: -1.8 to -0.72; p < 0.001), the Performance Intelligence Quotient (PIQ^[[55]](#footnote-55)^) (β = -1.4, 95%CI: -1.9 to -0.87; p < 0.001; β = -1.4; 95%CI: -1.9 to -0.80; p < 0.001), and FSIQ (β = -1.6; 95%CI: -2.1, -1.1; p < 0.001, and β = -1.4, 95% CI: -2.0 to -0.90; p < 0.001)  Urinary lead not associated with any of the outcomes  Urinary cadmium not associated with behavior  Maternal urinary arsenic > 524 µg/dL resulted in a 1.4-point decrease in FSIQ (95% CI: -2.7 to -0.03) and a 2.3 point decrease in VIQ (95% CI: -3.7 to -0.86), but resulted in a nonsignificant change in PIQ.  Concurrent urinary arsenic > 364 µg/dL resulted in a decrease of 2.1 points in VIQ (95% CI: -3.6 to -0.62) but resulted in nonsignificant changes in FSIQ and PIQ | Age, sex, weight, height, birth weight, birth order, socioeconomic status, parental education | Good |
| Marques, et al. (2014) [89] | Cohort | Brazil | n = 66, total population n = 51 living in the vicinity of tin-ore kilns and smelters  n = 45 living in Itapua, a fishing village | 6 and 24 months | No | Mercury, lead | Mercury: hair  Lead: breast milk | BSID-III | Mean (SD)  Tin-ore kilns and smelters Hair mercury: birth: 1.58 (0.91) µg/g Hair mercury, 6 months: 1.69 (0.86) µg/g Hair mercury, 24 months: 2.46 (1.08) µg/g Lead: 10.04 (8.37) µg/L  Itapua Hair mercury:, birth: 2.04 (0.99) µg/g Hair mercury, 6 months: 3.02 (1.11) µg/g Hair mercury, 24 months: 4.85 (1.64) µg/g Lead: 3.89 (3.78) µg/L | MDI at 6 months negatively associated with breast-milk lead (β = -0.293; 95% CI: -0.50, 0.08, p = 0.007). MDI at 24 months and PDI not significantly associated with lead levels  PDI at 6 months and MDI not significantly associated with mercury levels | Birth weight, presence of thiomersal containing vaccines, age of breast milk lead concentration, breastfeeding duration, family income, maternal schooling | Good |
| Menezes-Filho, et al. (2018) [90] | Cross-sectional | Brazil | n = 225 | 7-12 years | No | Lead, manganese | Lead: blood  Manganese: hair and toenail | Wechsler Abbreviated Scale of Intelligence | Median (range) Blood lead: .2 (0.3-15.6) µg/dL Hair manganese: 0.74 (0.16-8.79) µg/g Toenail manganese: 0.85 (0.15- 13.30) µg/g | At high toenail manganese, log blood lead levels negatively associated with IQ (t stat = -2.140, p < 0.036) At low toenail manganese levels, log blood lead levels not significantly associated with IQ (t stat = -1.380, p = 0.172)  Log hair manganese levels not correlated with IQ | Maternal IQ | Good |
| Mohamed, et al. (2015) [91] | Case control | Egypt | n = 200, total population n = 100 children with autism n = 100 children without autism as age and sex-matched controls, unrelated to the study group | 2.5-16 years | No | Lead, mercury, aluminum | Hair | Childhood Autism Rating Scale and Stanford-Binet Intelligence Scale | Mean (SD) ASD:  Lead 3.31 (3.92) mg/kg Mercury: 0.39 (0.37) mg/kg Aluminum: 59.19 (37.98) mg/kg  Controls:  Lead: 2.06 (2.45) mg/kg Mercury: 0.25 (0.16) mg/kg Aluminum: 16.78 (17.31) mg/kg | Lead, mercury, and aluminum significantly higher in children with ASD compared to controls (3.31± 3.92 mg/kg vs 2.06 ± 2.45 mg/kg; p= 0.015, 0.39 ± 0.37 mg/kg vs 0.25± 0.16; p = 0.023, and 59.19± 37.98 vs 16.78 ± 17.31 mg/kg; p = 0.0001, respectively) | Age, sex | Fair |
| Nyanza, et al (2021) [92] | Cohort | Tanzania | N = 439 | 6-12 months | Yes | Arsenic, cadmium, lead, mercury | Cadmium, lead, mercury: blood  Arsenic: urine | Malawi Developmental Assessment Tool | Median (IQR)  Arsenic: 8.3 (4.5-14.9) µg/L  Cadmium: 0.20 (0.2-0.3) µg/L  Lead: 27.2 (17.2-2.5) µg/dL  Mercury: 1.2 (0.8-1.7) µg/dL | Prenatal mercury inversely associated with global neurodevelopment (95% CI: 1.01-1.04; p < 0.001) and language impairment (95% CI: 1.03-1.07; p < 0.001)  When prenatal mercury and arsenic levels were above 0.80 µg/L and 15 µg/L respectively, the prevalence rate of neurodevelopmental impairment was two times higher (95% CI: 1.0-44.3, p = 0.034)  When prenatal mercury was above 0.8 µg/L and lead was at or above 35 µg/L, there was a 40% increase in neurodevelopmental impairment (95% CI: 0.9-2.1; p = 0.027) | Maternal age at birth, maternal education, maternal and paternal occupation, number of under-five siblings at home, family socioeconomic status, child sex, age, birthweight, height, weight | Good |
| Riojas-Rodriguez, et al. (2010) [93] | Cross-sectional | Mexico | n = 172, total population n = 79 living in Molango, a mining district n = 93, living in Agua Blanca | 7-11 years | No | Manganese, lead | Hair, blood | Weschler Intelligence Scale for Children, Revised | Mean (range)  Mining district Hair manganese: 12.13 (4.2-48.0) µg/g Blood manganese: 9.71 (5.5-18) µg/L Blood manganese: 7.96 (1.85-22.5) µg/dL  Non-mining district Hair manganese: 0.57 (0.06-3.6) µg/g Blood manganese: 9.71 (5.5-18) µg/L Blood lead: 3.37 (0.50-13.5) µg/dL | Hair manganese: Inversely associated with VIQ (β = -0.29; 95% CI: -0.51, -0.08), PIQ (β = -0.08, 95% CI: -0.32, 0.16), FSIQ (β = -0.20, 95%CI: -0.42, 0.02)  Blood manganese: not associated with VIQ (β = 0.69; 95% CI: -1.48, 0.09), PIQ (β = 0.02; 95% CI: -0.80, 0.83), or FSIQ (β = -0.4, 95% CI: -1.19, 0.39)  Lead: not associated with VIQ (β = 0.45; 95% CI: -0.10, 1.01), PIQ (β = -0.02; 95% CI: -0.63, 0.60), or FSIQ (β = 0.24; -0.32, 0.81) | Age, sex, nutritional status | Good |
| Rodrigues, et al. (2016) [94] | Cohort | Bangladesh | n = 525 total population n = 239, living in Pabna, a low exposure area n = 286, living in Sirajdikhan, a high exposure area | 20-40 months | Yes | Arsenic, manganese, lead | Arsenic: water  Manganese: water  Lead: blood | BSID-III | Median (IQR) Sirajdikhan Water arsenic First trimester: 1.3 (below the limit of detection (LOD^[[56]](#footnote-56)^)-1.9) µg/L 1 month: 0.8 (LOD-120) µg/L 12 months: 0.8 (LOD-38.2) µg/L 20-40 months: 1.5 (LOD-21.6) µg/L  Water manganese 1st trimester: 540 (30-960) µg/L 1 month: 560 (39-890) µg/L 12 months: 1150 (217-2000) µg/L 20-40 months: 948 (164-1820) µg/L  Blood lead 20-40 months: 7.6 (5.5- 10.4) µg/dL  Pabna Water arsenic First trimester: 26.5 (5.3-81) µg/L 1 month: 31 (5.9-120) µg/L 12 months: 30.5 (4.5-125) µg/L 20-40 months: 25.7 (4.4-130) µg/L  Water manganese 1st trimester: 500 (270-900) µg/L 1 month: 545 (340-1060) µg/L 12 months: 464 (244-891) µg/L 20-40 months: 515 (299-969) µg/L  Blood lead: 20-40 months: LOD (LOD- 3.8) µg/dL | Ln blood lead was associated with a lower cognitive score in Sirajdikhan (β = -0.17; SE: 0.09; p = 0.05). Water arsenic levels were associated with a lower cognitive score in Pabna (β -0.06; SE: 0.03; p = 0.05), and ln water manganese was associated with a reduced fine motor score in Pabna (β = -0.08; SE: 0.03; p = 0.02)  Median blood lead levels were higher in Sirajdikhan than Pabna (7.6 vs < LOD µg/dL, p< 0.0001) and water arsenic concentrations were lower (1.5 vs 25.7 µg/L, p < 0.0001) | Sex, maternal age, maternal education, exposure to environmental tobacco smoke, HOME^[[57]](#footnote-57)^ score, maternal Raven score | Good |
| Valeri, et al. (2017) [95] | Cohort | Bangladesh | n = 825 total population n = 409, living in Pabna, a a low exposure area n = 416, living in Sirajdikhan, a high exposure area | 20-40 months | No | Arsenic, manganese, lead | Blood | BSID-III | Mean (SD) Pabna Blood arsenic: 1.0 (2.2) µg/dL Blood manganese: 9.7 (2.6) µg/dL Blood lead: 1.8 (1.0) µg/dL  Sirajdikhan Blood arsenic: 0.4 (1.9) µg/dL Blood manganese: 5.4 (1.68) µg/dL Blood lead: 6.0 (1.89) µg/dL | In Pabna, manganese was negatively associated with the Cognitive score after adjusting for lead (β = -0.2, 95% CI: -0.4 to -0.02), but not the other metals  In Sirajdikhan, log lead concentration was marginally associated with lower Cognitive scores (β = -0.1, 95% CI: -0.2 to 0.0). Log arsenic concentration was associated with a lower Linguistic score (β = -0.08; 95% CI: -0.2 to 0.0)  In Pabna, children with blood metal concentrations above the 60th percentile (arsenic > 0.7 µg/dL, manganese > 4.2 µg/dL, lead > 6.6 µg/dL) had significantly lower BSID-III scores than those with median levels (data not published) | Sex, age, maternal age at time of birth, maternal IQ, HOME score, secondhand smoke exposure at baseline, protein intake | Good |
| Wasserman, et al. (2004) [96] | Cross-sectional | Bangladesh | n = 176 | 9.5- 10.5 years | No | Arsenic, manganese | Arsenic: Water, urine  Manganese: water | Wechsler Intelligence Scale for Children (WISC^[[58]](#footnote-58)^)-III | Mean (SD) Water manganese: 1386 (927) µg/L Water arsenic: 117.8 (145.2) µg/L Urinary arsenic: 116 (148.8) µg/L Blood lead: 10.1 (3.3) | Water arsenic: third (50.1-176 µg/L) and fourth quartiles (177-790 µg/L) had lower scores on PIQ (β = -7.3 and -9.7, p < 0.05, p< 0.01, respectively) and FSIQ (β = -7.8; p < 0.05 and β = 11.3, and <0.01, respectively) compared with lowest quartile (0.1-5.5 µg/L). Highest quartile slightly lower scores on VIQ than lowest quartile (β = -1.6, p < 0.10)  No correlation between water manganese and WISC scores (p > 0.25) | Age, sex, height, weight, head circumference, body mass index, television access, house type, maternal and paternal education, parental occupation, maternal age, maternal Raven’s score | Good |
| Wasserman, et al. (2006) [97] | Cross-sectional | Bangladesh | n = 142 | 10 years | No | Manganese, arsenic | Manganese: water, blood  Arsenic: Water, urine, blood  Lead: blood Chromium: urine | WISC-III | Mean (SD):  Water Manganese: 795 (755) µg/L Arsenic: 3.0 (2.6) µg/L  Urine Arsenic: 57.5 (67.6) µg/L Chromium 45.4 (30.2) mg/dL  Blood Manganese: 12.8 (3.2) µg/L Arsenic: 4.3 (1.9) µg/L Lead: 12.0 (3.7) µg/dL | Water manganese: negatively correlated with VIQ (B value: -0.63; p = 0.05), PIQ (B value = -3.76; p = 0.001), and FSIQ (B value: -4.35; p < 0.001)  Blood lead: negatively correlated with intelligence (data not published)  Water arsenic: no correlation with any of the subtests (data not published) | Age, sex, height, weight, head circumference, body mass index, television access, house type, maternal and paternal education, parental occupation, maternal age, maternal Raven’s score | Good |
| Wasserman, et al. (2007) [98] | Cross-sectional | Bangladesh | n = 301 | 6 years | No | Arsenic, manganese | Arsenic: Water, urine  Manganese: water  Lead: Blood | Wechsler Preschool and Primary Scale of Intelligence-III | Mean (SD) Water manganese: 1302 (821) µg/L Water arsenic: 120.1 (134.4) µg/L Urinary arsenic: 110.7 (132.8) µg/L Blood lead: 11.9 (3.5) µg/dL | Water arsenic: negatively correlated with performance (β = -0.48, p < 0.05), processing speed (β = -0.54, p < 0.05), and FSIQ raw scores (β = -1.06, p < 0.07); no correlation with verbal subtest (data not published)  Water manganese and blood lead not associated with IQ outcomes (data not published) | Age, sex, height, weight, head circumference, body mass index, television access, house type, maternal and paternal education, parental occupation, maternal age, maternal Raven’s score | Good |
| Wasserman, et al. (2011) [99] | Cross-sectional | Bangladesh | n = 303 | 8-11 years | No | Arsenic, manganese, lead, selenium | Arsenic: water, urine, blood  Manganese: Water, Blood  Lead: blood Selenium: blood | WISC-IV | Mean (SD) Arsenic: Water: 43.32 (73.65) µg/L Urine: 78.09 (72.16) µg/L Blood: 4.81 (3.22) µg/L  Manganese: Water: 725.54 (730.04) µg/L Blood: 4.81 (3.22) µg/L  Lead Blood: 114.56 (37.22) µg/L  Selenium: 104.97 (17.23) µg/L | Blood arsenic levels negatively correlated with verbal comprehension scores (β = -1.49, p < 0.05) but not with FSIQ, working memory, perceptual reasoning, or processing speed subtests   Blood manganese levels negatively correlated with working memory (β = -2.56, p< 0.01) and perceptual reasoning (β = -4.88, p< 0.01) subtests but not with FSIQ, verbal comprehension, or processing speed subtest | Age, sex, months attending school, body mass index, head circumference, maternal age, maternal education, home stimulation, hemoglobin | Good |
| Yu, et al, (2011) [100] | Cohort | China | n = 1652 | 3 days old | No | Lead, mercury, cadmium, arsenic, thallium | Lead, mercury: blood  Cadmium, arsenic, thallium: serum | Neonatal behavioral neurological assessments (NBNA^[[59]](#footnote-59)^) | Median (range) Lead: 41.00 (5.00-351.00) µg/L Mercury: 1.88 (0.18-13.24) µg/L Cadmium: 0.03 (no data- 6.46) µg/L Arsenic: 0.86 (ND - 375.50) µg/L Thallium: 0.02 (ND- 0.15) µg/L | NBNA scores in children with mercury levels > 5.8 µg/L were lower than children with mercury <2.9 µg/L (38.50 ± 1.63 vs 39.11 ± 1.30, respectively; p < 0.05)  NBNA scores in children with cadmium levels > 0.37 µg/L were lower than children with cadmium levels < 0.18 µg/L (37.88 ± 1.63 vs 39.38 ± 1.04 µg/L, respectively; p < 0.05)  NBNA scores in children with thallium levels> 0.022 µg/L were lower than children with thallium < 0.011 µg/L (38.65 ± 1.51 vs 39.49 ± 0.87 µg/L, respectively; p < 0.05)  NBNA scores in children with arsenic levels between 30 and 50 µg/L were lower than children with arsenic levels < 30 µg/L (37.80 ± 1.30 vs 39.13 ± 1.28, respectively; p< 0.05)  NBNA scores were not associated with lead levels | Sex, maternal age, maternal education level, maternal occupation, monthly household income, presence of smoking, type of fish intake, presence of egg and popcorn intake during pregnancy, presence of prenatal care, infection history during pregnancy | Good |

Works cited

1. Asadullah MN, Chaudhury N. Poisoning the mind: Arsenic contamination of drinking water wells and children’s educational achievement in rural Bangladesh. Economics of Education Review. 2011;30: 873–888. doi:10.1016/j.econedurev.2011.05.001

2. Hamadani JD, Grantham-McGregor SM, Tofail F, Nermell B, Fängström B, Huda SN, et al. Pre- and postnatal arsenic exposure and child development at 18 months of age: a cohort study in rural Bangladesh. Int J Epidemiol. 2010;39: 1206–1216. doi:10.1093/ije/dyp369

3. Hamadani JD, Tofail F, Nermell B, Gardner R, Shiraji S, Bottai M, et al. Critical windows of exposure for arsenic-associated impairment of cognitive function in pre-school girls and boys: a population-based cohort study. Int J Epidemiol. 2011;40: 1593–1604. doi:10.1093/ije/dyr176

4. Rocha-Amador D, Navarro ME, Carrizales L, Morales R, Calderón J. Decreased intelligence in children and exposure to fluoride and arsenic in drinking water. Cadernos de Saúde Pública. 2007;23: S579–S587. doi:10.1590/S0102-311X2007001600018

5. Rosado JL, Ronquillo D, Kordas K, Rojas O, Alatorre J, Lopez P, et al. Arsenic Exposure and Cognitive Performance in Mexican Schoolchildren. Environ Health Perspect. 2007;115: 1371–1375. doi:10.1289/ehp.9961

6. Roy A, Kordas K, Lopez P, Rosado JL, Cebrian ME, Vargas GG, et al. Association between arsenic exposure and behavior among first-graders from Torreón, Mexico. Environ Res. 2011;111: 670–676. doi:10.1016/j.envres.2011.03.003

7. Siripitayakunkit U, Visudhiphan P, Pradipasen M, Vorapongsathron T. Association between Chronic Arsenic Exposure and Children’s Intelligence in Thailand. In: Chappell WR, Abernathy CO, Calderon RL, editors. Arsenic Exposure and Health Effects III. Oxford: Elsevier Science Ltd; 1999. pp. 141–149. doi:10.1016/B978-008043648-7/50018-2

8. Tofail F, Vahter M, Hamadani JD, Nermell B, Huda SN, Yunus M, et al. Effect of Arsenic Exposure during Pregnancy on Infant Development at 7 Months in Rural Matlab, Bangladesh. Environ Health Perspect. 2009;117: 288–293. doi:10.1289/ehp.11670

9. Vahter M, Skröder H, Rahman SM, Levi M, Derakhshani Hamadani J, Kippler M. Prenatal and childhood arsenic exposure through drinking water and food and cognitive abilities at 10 years of age: A prospective cohort study. Environment International. 2020;139: 105723. doi:10.1016/j.envint.2020.105723

10. von Ehrenstein OS, Poddar S, Yuan Y, Mazumder DG, Eskenazi B, Basu A, et al. Children’s intellectual function in relation to arsenic exposure. Epidemiology. 2007;18: 44–51. doi:10.1097/01.ede.0000248900.65613.a9

11. Wang S-X, Wang Z-H, Cheng X-T, Li J, Sang Z-P, Zhang X-D, et al. Arsenic and fluoride exposure in drinking water: children’s IQ and growth in Shanyin county, Shanxi province, China. Environ Health Perspect. 2007;115: 643–647. doi:10.1289/ehp.9270

12. Gustin K, Tofail F, Vahter M, Kippler M. Cadmium exposure and cognitive abilities and behavior at 10 years of age: A prospective cohort study. Environ Int. 2018;113: 259–268. doi:10.1016/j.envint.2018.02.020

13. Tian L-L, Zhao Y-C, Wang X-C, Gu J-L, Sun Z-J, Zhang Y-L, et al. Effects of Gestational Cadmium Exposure on Pregnancy Outcome and Development in the Offspring at Age 4.5 Years. Biol Trace Elem Res. 2009;132: 51–59. doi:10.1007/s12011-009-8391-0

14. Wang Y, Chen L, Gao Y, Zhang Y, Wang C, Zhou Y, et al. Effects of prenatal exposure to cadmium on neurodevelopment of infants in Shandong, China. Environ Pollut. 2016;211: 67–73. doi:10.1016/j.envpol.2015.12.038

15. AbuShady MM, Fathy HA, Fathy GA, Fatah SAE, Ali A, Abbas MA. Blood lead levels in a group of children: the potential risk factors and health problems. J Pediatr (Rio J). 2017;93: 619–624. doi:10.1016/j.jped.2016.12.006

16. Alvarez-Ortega N, Caballero-Gallardo K, Olivero-Verbel J. Low blood lead levels impair intellectual and hematological function in children from Cartagena, Caribbean coast of Colombia. J Trace Elem Med Biol. 2017;44: 233–240. doi:10.1016/j.jtemb.2017.08.006

17. Bellinger DC, Hu H, Kalaniti K, Thomas N, Rajan P, Sambandam S, et al. A pilot study of blood lead levels and neurobehavioral function in children living in Chennai, India. Int J Occup Environ Health. 2005;11: 138–143. doi:10.1179/oeh.2005.11.2.138

18. Braun JM, Hoffman E, Schwartz J, Sanchez B, Schnaas L, Mercado-Garcia A, et al. Assessing windows of susceptibility to lead-induced cognitive deficits in Mexican children. Neurotoxicology. 2012;33: 1040–1047. doi:10.1016/j.neuro.2012.04.022

19. Counter SA, Buchanan LH, Rosas HD, Ortega F. Neurocognitive effects of chronic lead intoxication in Andean children. J Neurol Sci. 1998;160: 47–53. doi:10.1016/s0022-510x(98)00180-4

20. Counter SA, Buchanan LH, Ortega F. Neurocognitive impairment in lead-exposed children of Andean lead-glazing workers. J Occup Environ Med. 2005;47: 306–312. doi:10.1097/01.jom.0000155717.45594.65

21. Counter SA, Buchanan LH, Ortega F. Zinc protoporphyrin levels, blood lead levels and neurocognitive deficits in Andean children with chronic lead exposure. Clin Biochem. 2008;41: 41–47. doi:10.1016/j.clinbiochem.2007.10.002

22. do Nascimento SN, Charão MF, Moro AM, Roehrs M, Paniz C, Baierle M, et al. Evaluation of Toxic Metals and Essential Elements in Children with Learning Disabilities from a Rural Area of Southern Brazil. Int J Environ Res Public Health. 2014;11: 10806–10823. doi:10.3390/ijerph111010806

23. Gahyva DLC, Crenitte P de AP, Caldana M de L, Hage SR de V. Characterization of language disorders in children with lead poisoning. Pro Fono. 2008;20: 55–60. doi:10.1590/s0104-56872008000100010

24. Gleason KM, Valeri L, Shankar AH, Obrycki JF, Ibne Hasan MOS, Mostofa G, et al. Stunting and lead: using causal mediation analysis to better understand how environmental lead exposure affects cognitive outcomes in children. J Neurodev Disord. 2020;12. doi:10.1186/s11689-020-09346-x

25. Kamel NM, Ramadan AM, Kamel MI, Mostafa YAEG, Abo el-Naga RM, Ali AM. Impact of lead exposure on health status and scholastic achievement of school pupils in Alexandria. J Egypt Public Health Assoc. 2003;78: 1–28.

26. Kashala-Abotnes E, Mumbere PP, Mishika JM, Ndjukendi AO, Mpaka DB, Bumoko M-MG, et al. Lead exposure and early child neurodevelopment among children 12-24 months in Kinshasa, the Democratic Republic of Congo. Eur Child Adolesc Psychiatry. 2016;25: 1361–1367. doi:10.1007/s00787-016-0860-3

27. Kordas K, Lopez P, Rosado JL, García Vargas G, Alatorre Rico J, Ronquillo D, et al. Blood Lead, Anemia, and Short Stature Are Independently Associated with Cognitive Performance in Mexican School Children. J Nutr. 2004;134: 363–371. doi:10.1093/jn/134.2.363

28. Kordas K, Canfield RL, López P, Rosado JL, Vargas GG, Cebrián ME, et al. Deficits in cognitive function and achievement in Mexican first-graders with low blood lead concentrations. Environ Res. 2006;100: 371–386. doi:10.1016/j.envres.2005.07.007

29. Kordas K, Ettinger AS, Bellinger DC, Schnaas L, Téllez Rojo MM, Hernández-Avila M, et al. A dopamine receptor (DRD2) but not dopamine transporter (DAT1) gene polymorphism is associated with neurocognitive development of Mexican preschool children with lead exposure. J Pediatr. 2011;159: 638–643. doi:10.1016/j.jpeds.2011.03.043

30. L M, Mitra P, Goyal T, Sharma S, Purohit P, Sharma P. Association of blood lead levels with neurobehavior and BDNF expression in school going children. J Trace Elem Med Biol. 2021;66: 126749. doi:10.1016/j.jtemb.2021.126749

31. Liu J, Li L, Wang Y, Yan C, Liu X. Impact of low blood lead concentrations on IQ and school performance in Chinese children. PLoS ONE. 2013;8: e65230. doi:10.1371/journal.pone.0065230

32. Mostafa GA, El‐Shahawi HH, Mokhtar A. Blood lead levels in Egyptian children from high and low lead-polluted areas: impact on cognitive function. Acta Neurologica Scandinavica. 2009;120: 30–37. doi:10.1111/j.1600-0404.2009.01155.x

33. Olympio KPK, Oliveira PV, Naozuka J, Cardoso MRA, Marques AF, Günther WMR, et al. Surface dental enamel lead levels and antisocial behavior in Brazilian adolescents. Neurotoxicol Teratol. 2010;32: 273–279. doi:10.1016/j.ntt.2009.12.003

34. Rahbar MH, Samms-Vaughan M, Dickerson AS, Loveland KA, Ardjomand-Hessabi M, Bressler J, et al. Blood Lead Concentrations in Jamaican Children with and without Autism Spectrum Disorder. Int J Environ Res Public Health. 2015;12: 83–105. doi:10.3390/ijerph120100083

35. Rahman A, Maqbool E, Zuberi HS. Lead-associated deficits in stature, mental ability and behaviour in children in Karachi. Ann Trop Paediatr. 2002;22: 301–311. doi:10.1179/027249302125001958

36. Rasoul GA, Al-Batanony MA, Mahrous OA, Abo-Salem ME, Gabr HM. Environmental Lead Exposure among Primary School Children in Shebin El-Kom District, Menoufiya Governorate, Egypt. Int J Occup Environ Med (The IJOEM). 2012;3. Available: https://www.theijoem.com/ijoem/index.php/ijoem/article/view/155

37. Ruiz-Castell M, Paco P, Barbieri F-L, Duprey J-L, Forns J, Carsin A-E, et al. Child neurodevelopment in a Bolivian mining city. Environmental Research. 2012;112: 147–154. doi:10.1016/j.envres.2011.12.001

38. Schnaas L, Rothenberg SJ, Perroni E, Martínez S, Hernández C, Hernández RM. Temporal pattern in the effect of postnatal blood lead level on intellectual development of young children. Neurotoxicol Teratol. 2000;22: 805–810. doi:10.1016/s0892-0362(00)00101-x

39. Schnaas L, Rothenberg SJ, Flores M-F, Martinez S, Hernandez C, Osorio E, et al. Reduced Intellectual Development in Children with Prenatal Lead Exposure. Environ Health Perspect. 2006;114: 791–797. doi:10.1289/ehp.8552

40. Solon O, Riddell TJ, Quimbo SA, Butrick E, Aylward GP, Lou Bacate M, et al. Associations between Cognitive Function, Blood Lead Concentration, and Nutrition among Children in the Central Philippines. The Journal of Pediatrics. 2008;152: 237-243.e1. doi:10.1016/j.jpeds.2007.09.008

41. Téllez-Rojo MM, Bellinger DC, Arroyo-Quiroz C, Lamadrid-Figueroa H, Mercado-García A, Schnaas-Arrieta L, et al. Longitudinal associations between blood lead concentrations lower than 10 microg/dL and neurobehavioral development in environmentally exposed children in Mexico City. Pediatrics. 2006;118: e323-330. doi:10.1542/peds.2005-3123

42. Vega-Dienstmaier JM, Salinas-Piélago JE, Gutiérrez-Campos M del R, Mandamiento-Ayquipa RD, Yara-Hokama M del C, Ponce-Canchihuamán J, et al. Lead levels and cognitive abilities in Peruvian children. Braz J Psychiatry. 2006;28: 33–39. doi:10.1590/s1516-44462006000100008

43. Vigeh M, Yokoyama K, Matsukawa T, Shinohara A, Ohtani K. Low level prenatal blood lead adversely affects early childhood mental development. J Child Neurol. 2014;29: 1305–1311. doi:10.1177/0883073813516999

44. Wang L, Xu SE, Zhang GD, Wang WY. Study of lead absorption and its effect on children’s development. Biomed Environ Sci. 1989;2: 325–330.

45. Wang Q, Zhao HH, Chen JW, Gu KD, Zhang YZ, Zhu YX, et al. Adverse health effects of lead exposure on children and exploration to internal lead indicator. Sci Total Environ. 2009;407: 5986–5992. doi:10.1016/j.scitotenv.2009.08.038

46. Wasserman GA, Staghezza-Jaramillo B, Shrout P, Popovac D, Graziano J. The effect of lead exposure on behavior problems in preschool children. Am J Public Health. 1998;88: 481–486.

47. Wasserman GA, Factor-Litvak P, Liu X, Todd AC, Kline JK, Slavkovich V, et al. The relationship between blood lead, bone lead and child intelligence. Child Neuropsychol. 2003;9: 22–34. doi:10.1076/chin.9.1.22.14497

48. Wolf AW, Jimenez E, Lozoff B. No evidence of developmental III effects of low-level lead exposure in a developing country. J Dev Behav Pediatr. 1994;15: 224–231.

49. Betancourt Ó, Tapia M, Méndez I. Decline of General Intelligence in Children Exposed to Manganese from Mining Contamination in Puyango River Basin, Southern Ecuador. Ecohealth. 2015;12: 453–460. doi:10.1007/s10393-015-1027-2

50. Carvalho CF, Menezes-Filho JA, de Matos VP, Bessa JR, Coelho-Santos J, Viana GFS, et al. Elevated airborne manganese and low executive function in school-aged children in Brazil. Neurotoxicology. 2014;45: 301–308. doi:10.1016/j.neuro.2013.11.006

51. Carvalho CF de, Oulhote Y, Martorelli M, Carvalho CO de, Menezes-Filho JA, Argollo N, et al. Environmental manganese exposure and associations with memory, executive functions, and hyperactivity in Brazilian children. Neurotoxicology. 2018;69: 253–259. doi:10.1016/j.neuro.2018.02.002

52. Claus Henn B, Ettinger AS, Schwartz J, Téllez-Rojo MM, Lamadrid-Figueroa H, Hernández-Avila M, et al. Early postnatal blood manganese levels and children’s neurodevelopment. Epidemiology. 2010;21: 433–439. doi:10.1097/ede.0b013e3181df8e52

53. Menezes-Filho JA, de Carvalho-Vivas CF, Viana GFS, Ferreira JRD, Nunes LS, Mergler D, et al. Elevated manganese exposure and school-aged children’s behavior: A gender-stratified analysis. NeuroToxicology. 2014;45: 293–300. doi:10.1016/j.neuro.2013.09.006

54. Menezes-Filho JA, Novaes C de O, Moreira JC, Sarcinelli PN, Mergler D. Elevated manganese and cognitive performance in school-aged children and their mothers. Environ Res. 2011;111: 156–163. doi:10.1016/j.envres.2010.09.006

55. Nascimento S, Baierle M, Göethel G, Barth A, Brucker N, Charão M, et al. Associations among environmental exposure to manganese, neuropsychological performance, oxidative damage and kidney biomarkers in children. Environ Res. 2016;147: 32–43. doi:10.1016/j.envres.2016.01.035

56. Rahbar MH, Samms-Vaughan M, Dickerson AS, Loveland KA, Ardjomand-Hessabi M, Bressler J, et al. Blood manganese concentrations in Jamaican children with and without autism spectrum disorders. Environ Health. 2014;13: 69. doi:10.1186/1476-069X-13-69

57. Rahman SM, Kippler M, Tofail F, Bölte S, Derakhshani Hamadani J, Vahter M. Manganese in Drinking Water and Cognitive Abilities and Behavior at 10 Years of Age: A Prospective Cohort Study. Environ Health Perspect. 2017;125. doi:10.1289/EHP631

58. Torres-Agustín R, Rodríguez-Agudelo Y, Schilmann A, Solís-Vivanco R, Montes S, Riojas-Rodríguez H, et al. Effect of environmental manganese exposure on verbal learning and memory in Mexican children. Environ Res. 2013;121: 39–44. doi:10.1016/j.envres.2012.10.007

59. Yu X-D, Zhang J, Yan C-H, Shen X-M. Prenatal exposure to manganese at environment relevant level and neonatal neurobehavioral development. Environ Res. 2014;133: 232–238. doi:10.1016/j.envres.2014.04.012

60. Chevrier C, Sullivan K, White RF, Comtois C, Cordier S, Grandjean P. Qualitative assessment of visuospatial errors in mercury-exposed Amazonian children. Neurotoxicology. 2009;30: 37–46. doi:10.1016/j.neuro.2008.09.012

61. Counter SA, Buchanan LH, Ortega F. Neurocognitive screening of mercury-exposed children of Andean gold miners. Int J Occup Environ Health. 2006;12: 209–214. doi:10.1179/oeh.2006.12.3.209

62. Dórea JG, Marques RC, Isejima C. Neurodevelopment of Amazonian Infants: Antenatal and Postnatal Exposure to Methyl- and Ethylmercury. In: Journal of Biomedicine and Biotechnology [Internet]. Hindawi; 26 Apr 2012 [cited 29 Jun 2020] p. e132876. doi:https://doi.org/10.1155/2012/132876

63. Dórea JG, Marques RC, Abreu L. Milestone achievement and neurodevelopment of rural Amazonian toddlers (12 to 24 months) with different methylmercury and ethylmercury exposure. J Toxicol Environ Health Part A. 2014;77: 1–13. doi:10.1080/15287394.2014.861335

64. Fonseca M de F, Dórea JG, Bastos WR, Marques RC, Torres JPM, Malm O. Poor psychometric scores of children living in isolated riverine and agrarian communities and fish-methylmercury exposure. Neurotoxicology. 2008;29: 1008–1015. doi:10.1016/j.neuro.2008.07.001

65. Gao Y, Yan C-H, Tian Y, Wang Y, Xie H-F, Zhou X, et al. Prenatal exposure to mercury and neurobehavioral development of neonates in Zhoushan City, China. Environmental Research. 2007;105: 390–399. doi:10.1016/j.envres.2007.05.015

66. Grandjean P, White RF, Nielsen A, Cleary D, de Oliveira Santos EC. Methylmercury neurotoxicity in Amazonian children downstream from gold mining. Environ Health Perspect. 1999;107: 587–591.

67. Gustin K, Tofail F, Mehrin F, Levi M, Vahter M, Kippler M. Methylmercury exposure and cognitive abilities and behavior at 10years of age. Environ Int. 2017;102: 97–105. doi:10.1016/j.envint.2017.02.004

68. Hu Y, Chen L, Wang C, Zhou Y, Zhang Y, Wang Y, et al. Prenatal low-level mercury exposure and infant neurodevelopment at 12 months in rural northern China. Environ Sci Pollut Res Int. 2016;23: 12050–12059. doi:10.1007/s11356-016-6395-9

69. Marques RC, Abreu L, Bernardi JVE, Dórea JG. Neurodevelopment of Amazonian children exposed to ethylmercury (from Thimerosal in vaccines) and methylmercury (from fish). Environ Res. 2016;149: 259–265. doi:10.1016/j.envres.2015.12.022

70. Marques RC, Garrofe Dórea J, Rodrigues Bastos W, de Freitas Rebelo M, de Freitas Fonseca M, Malm O. Maternal mercury exposure and neuro-motor development in breastfed infants from Porto Velho (Amazon), Brazil. Int J Hyg Environ Health. 2007;210: 51–60. doi:10.1016/j.ijheh.2006.08.001

71. Marques RC, Dórea JG, Bernardi JVE, Bastos WR, Malm O. Prenatal and postnatal mercury exposure, breastfeeding and neurodevelopment during the first 5 years. Cogn Behav Neurol. 2009;22: 134–141. doi:10.1097/WNN.0b013e3181a72248

72. Marques RC, Dórea JG, Bernardi JVE. Thimerosal exposure (from tetanus-diphtheria vaccine) during pregnancy and neurodevelopment of breastfed infants at 6 months. Acta Paediatr. 2010;99: 934–939. doi:10.1111/j.1651-2227.2009.01641.x

73. Marques RC, Dórea JG, Leão RS, Dos Santos VG, Bueno L, Marques RC, et al. Role of methylmercury exposure (from fish consumption) on growth and neurodevelopment of children under 5 years of age living in a transitioning (tin-mining) area of the western Amazon, Brazil. Arch Environ Contam Toxicol. 2012;62: 341–350. doi:10.1007/s00244-011-9697-4

74. Marques RC, Bernardi JVE, Abreu L, Dórea JG. Neurodevelopment outcomes in children exposed to organic mercury from multiple sources in a tin-ore mine environment in Brazil. Arch Environ Contam Toxicol. 2015;68: 432–441. doi:10.1007/s00244-014-0103-x

75. Marques RC, Bernardi JVE, Cunha MPL, Dórea JG. Impact of organic mercury exposure and home delivery on neurodevelopment of Amazonian children. International Journal of Hygiene and Environmental Health. 2016;219: 498–502. doi:10.1016/j.ijheh.2016.05.002

76. Marques RC, Abreu L, Bernardi JVE, Dórea JG. Traditional living in the Amazon: Extended breastfeeding, fish consumption, mercury exposure and neurodevelopment. Ann Hum Biol. 2016;43: 360–370. doi:10.1080/03014460.2016.1189962

77. Rothenberg SE, Korrick SA, Liu J, Nong Y, Nong H, Hong C, et al. Maternal methylmercury exposure through rice ingestion and child neurodevelopment in the first three years: a prospective cohort study in rural China. Environ Health. 2021;20: 50. doi:10.1186/s12940-021-00732-z

78. Tavares LMB, Câmara VM, Malm O, Santos EC de O. Performance on neurological development tests by riverine children with moderate mercury exposure in Amazonia, Brazil. Cadernos de Saúde Pública. 2005;21: 1160–1167. doi:10.1590/S0102-311X2005000400018

79. Bao Q-S, Lu C-Y, Song H, Wang M, Ling W, Chen W-Q, et al. Behavioural development of school-aged children who live around a multi-metal sulphide mine in Guangdong province, China: a cross-sectional study. BMC Public Health. 2009;9: 217. doi:10.1186/1471-2458-9-217

80. Bora BK, Ramos-Crawford AL, Sikorskii A, Boivin MJ, Lez DM, Mumba-Ngoyi D, et al. Concurrent exposure to heavy metals and cognition in school-age children in Congo-Kinshasa: A complex overdue research agenda. Brain Res Bull. 2019;145: 81–86. doi:10.1016/j.brainresbull.2018.06.013

81. Calderón J, Navarro ME, Jimenez-Capdeville ME, Santos-Diaz MA, Golden A, Rodriguez-Leyva I, et al. Exposure to Arsenic and Lead and Neuropsychological Development in Mexican Children. Environmental Research. 2001;85: 69–76. doi:10.1006/enrs.2000.4106

82. Claus Henn B, Schnaas L, Ettinger AS, Schwartz J, Lamadrid-Figueroa H, Hernández-Avila M, et al. Associations of early childhood manganese and lead coexposure with neurodevelopment. Environ Health Perspect. 2012;120: 126–131. doi:10.1289/ehp.1003300

83. do Nascimento SN, Barth A, Göethel G, Baierle M, Charão MF, Brucker N, et al. Cognitive deficits and ALA-D-inhibition in children exposed to multiple metals. Environmental Research. 2015;136: 387–395. doi:10.1016/j.envres.2014.10.003

84. Hernández-Bonilla D, Escamilla-Núñez C, Mergler D, Rodríguez-Dozal S, Cortez-Lugo M, Montes S, et al. Effects of manganese exposure on visuoperception and visual memory in schoolchildren. Neurotoxicology. 2016;57: 230–240. doi:10.1016/j.neuro.2016.10.006

85. Khaled EM, Meguid NA, Bjørklund G, Gouda A, Bahary MH, Hashish A, et al. Altered urinary porphyrins and mercury exposure as biomarkers for autism severity in Egyptian children with autism spectrum disorder. Metab Brain Dis. 2016;31: 1419–1426. doi:10.1007/s11011-016-9870-6

86. Khan K, Factor-Litvak P, Wasserman GA, Liu X, Ahmed E, Parvez F, et al. Manganese Exposure from Drinking Water and Children’s Classroom Behavior in Bangladesh. Environ Health Perspect. 2011;119: 1501–1506. doi:10.1289/ehp.1003397

87. Khan K, Wasserman GA, Liu X, Ahmed E, Parvez F, Slavkovich V, et al. Manganese exposure from drinking water and children’s academic achievement. Neurotoxicology. 2012;33: 91–97. doi:10.1016/j.neuro.2011.12.002

88. Kippler M, Tofail F, Hamadani JD, Gardner RM, Grantham-McGregor SM, Bottai M, et al. Early-life cadmium exposure and child development in 5-year-old girls and boys: a cohort study in rural Bangladesh. Environ Health Perspect. 2012;120: 1462–1468. doi:10.1289/ehp.1104431

89. Marques RC, Bernardi JVE, Dórea JG, de Fatima R Moreira M, Malm O. Perinatal multiple exposure to neurotoxic (lead, methylmercury, ethylmercury, and aluminum) substances and neurodevelopment at six and 24 months of age. Environ Pollut. 2014;187: 130–135. doi:10.1016/j.envpol.2014.01.004

90. Menezes-Filho JA, Carvalho CF, Rodrigues JLG, Araújo CFS, dos Santos NR, Lima CS, et al. Environmental Co-Exposure to Lead and Manganese and Intellectual Deficit in School-Aged Children. Int J Environ Res Public Health. 2018;15. doi:10.3390/ijerph15112418

91. Mohamed FEB, Zaky EA, El-Sayed AB, Elhossieny RM, Zahra SS, Salah Eldin W, et al. Assessment of Hair Aluminum, Lead, and Mercury in a Sample of Autistic Egyptian Children: Environmental Risk Factors of Heavy Metals in Autism. In: Behavioural Neurology [Internet]. Hindawi; 5 Oct 2015 [cited 2 Jul 2020] p. e545674. doi:https://doi.org/10.1155/2015/545674

92. Nyanza EC, Bernier FP, Martin JW, Manyama M, Hatfield J, Dewey D. Effects of prenatal exposure and co-exposure to metallic or metalloid elements on early infant neurodevelopmental outcomes in areas with small-scale gold mining activities in Northern Tanzania. Environ Int. 2021;149: 106104. doi:10.1016/j.envint.2020.106104

93. Riojas-Rodríguez H, Solís-Vivanco R, Schilmann A, Montes S, Rodríguez S, Ríos C, et al. Intellectual function in Mexican children living in a mining area and environmentally exposed to manganese. Environ Health Perspect. 2010;118: 1465–1470. doi:10.1289/ehp.0901229

94. Rodrigues EG, Bellinger DC, Valeri L, Hasan MOSI, Quamruzzaman Q, Golam M, et al. Neurodevelopmental outcomes among 2- to 3-year-old children in Bangladesh with elevated blood lead and exposure to arsenic and manganese in drinking water. Environmental Health. 2016;15: 44. doi:10.1186/s12940-016-0127-y

95. Valeri L, Mazumdar MM, Bobb JF, Claus Henn B, Rodrigues E, Sharif OIA, et al. The Joint Effect of Prenatal Exposure to Metal Mixtures on Neurodevelopmental Outcomes at 20-40 Months of Age: Evidence from Rural Bangladesh. Environ Health Perspect. 2017;125: 067015. doi:10.1289/EHP614

96. Wasserman GA, Liu X, Parvez F, Ahsan H, Factor-Litvak P, van Geen A, et al. Water Arsenic Exposure and Children’s Intellectual Function in Araihazar, Bangladesh. Environ Health Perspect. 2004;112: 1329–1333. doi:10.1289/ehp.6964

97. Wasserman GA, Liu X, Parvez F, Ahsan H, Levy D, Factor-Litvak P, et al. Water manganese exposure and children’s intellectual function in Araihazar, Bangladesh. Environ Health Perspect. 2006;114: 124–129. doi:10.1289/ehp.8030

98. Wasserman GA, Liu X, Parvez F, Ahsan H, Factor-Litvak P, Kline J, et al. Water Arsenic Exposure and Intellectual Function in 6-Year-Old Children in Araihazar, Bangladesh. Environ Health Perspect. 2007;115: 285–289. doi:10.1289/ehp.9501

99. Wasserman GA, Liu X, Parvez F, Factor-Litvak P, Ahsan H, Levy D, et al. Arsenic and manganese exposure and children’s intellectual function. Neurotoxicology. 2011;32: 450–457. doi:10.1016/j.neuro.2011.03.009

100. Yu X-D, Yan C-H, Shen X-M, Tian Y, Cao L-L, Yu X-G, et al. Prenatal exposure to multiple toxic heavy metals and neonatal neurobehavioral development in Shanghai, China. Neurotoxicol Teratol. 2011;33: 437–443. doi:10.1016/j.ntt.2011.05.010

1. IQR: Interquartile range [↑](#footnote-ref-1)
2. GW: Gestational week [↑](#footnote-ref-2)
3. HOME: Home Observation for Measurement of Environment [↑](#footnote-ref-3)
4. VIQ: Verbal Intelligence Quotient [↑](#footnote-ref-4)
5. CI: Confidence interval [↑](#footnote-ref-5)
6. FSIQ: Full Scale Intelligence Quotient [↑](#footnote-ref-6)
7. WISC-RM: Wechsler Intelligence Scale for Children, Revised Mexican Version [↑](#footnote-ref-7)
8. SD: Standard deviation [↑](#footnote-ref-8)
9. WISC: Wechsler’s Intelligence Scale for Children [↑](#footnote-ref-9)
10. CI: Confidence interval [↑](#footnote-ref-10)
11. SD: Standard deviation [↑](#footnote-ref-11)
12. CI: Confidence interval [↑](#footnote-ref-12)
13. RCPM: Raven’s Colored Progressive Matrices [↑](#footnote-ref-13)
14. WISC: Wechsler Intelligence Scale for Children [↑](#footnote-ref-14)
15. HOME: Home Observation for Measurement of Environment [↑](#footnote-ref-15)
16. WISC-RM: Wechsler Intelligence Scale for Children, Revised Mexican Version [↑](#footnote-ref-16)
17. BSID: Bayley Scales of Infant Development [↑](#footnote-ref-17)
18. MDI: Mental Developmental Index [↑](#footnote-ref-18)
19. PDI: Psychomotor Developmental Index [↑](#footnote-ref-19)
20. GCI: General Cognitive Index [↑](#footnote-ref-20)
21. WPPSI: Wechsler Preschool and Primary Scale of Intelligence [↑](#footnote-ref-21)
22. PIQ: Performance Intelligence Quotient [↑](#footnote-ref-22)
23. VIQ: Verbal Intelligence Quotient [↑](#footnote-ref-23)
24. FSIQ: Full Scale Intelligence Quotient [↑](#footnote-ref-24)
25. ASD: Autism Spectrum Disorder [↑](#footnote-ref-25)
26. HOME: Home Observation for Measurement of Environment [↑](#footnote-ref-26)
27. RCPM: Raven’s Colored Progressive Matrices [↑](#footnote-ref-27)
28. SD: Standard deviation [↑](#footnote-ref-28)
29. WISC: Wechsler Intelligence Scale for Children [↑](#footnote-ref-29)
30. CI: Confidence interval [↑](#footnote-ref-30)
31. HOME: Home Observation for Measurement of Environment [↑](#footnote-ref-31)
32. ASD: Autism Spectrum Disorder [↑](#footnote-ref-32)
33. OR: Odds ratio [↑](#footnote-ref-33)
34. NBNA: Neonatal behavioral neurological assessments [↑](#footnote-ref-34)
35. SD: Standard deviation [↑](#footnote-ref-35)
36. GDS: Gesell Developmental Schedules [↑](#footnote-ref-36)
37. WISC: Wechsler Intelligence Scale for Children [↑](#footnote-ref-37)
38. IQR: Interquartile range [↑](#footnote-ref-38)
39. OR: Odds ratio [↑](#footnote-ref-39)
40. CI: Confidence interval [↑](#footnote-ref-40)
41. HOME: Home Observation for Measurement of Environment [↑](#footnote-ref-41)
42. BSID: Bayley Scales of Infant Development [↑](#footnote-ref-42)
43. MDI: Mental Developmental Index [↑](#footnote-ref-43)
44. PDI: Psychomotor Developmental Index [↑](#footnote-ref-44)
45. SE: Standard error [↑](#footnote-ref-45)
46. SD: Standard deviation [↑](#footnote-ref-46)
47. VIQ: Verbal Intelligence Quotient [↑](#footnote-ref-47)
48. FSIQ: Full Scale Intelligence Quotient [↑](#footnote-ref-48)
49. BSID: Bayley Scales of Infant Development [↑](#footnote-ref-49)
50. MDI: Mental Developmental Index [↑](#footnote-ref-50)
51. CI: Confidence interval [↑](#footnote-ref-51)
52. PDI: Psychomotor Developmental Index [↑](#footnote-ref-52)
53. RCPM: Raven’s Colored Progressive Matrices [↑](#footnote-ref-53)
54. ASD: Autism Spectrum Disorder [↑](#footnote-ref-54)
55. PIQ: Performance Intelligence Quotient [↑](#footnote-ref-55)
56. LOD: Below the limit of detection [↑](#footnote-ref-56)
57. HOME: Home Observation for Measurement of Environment [↑](#footnote-ref-57)
58. WISC: Wechsler Intelligence Scale for Children [↑](#footnote-ref-58)
59. NBNA: Neonatal behavioral neurological assessments [↑](#footnote-ref-59)
